# Coffee endophytes: diversity, ecological functions, and application prospects in sustainable production

**DOI:** 10.3389/fpls.2026.1884416

**Published:** 2026-07-07

**Authors:** Qilong Wei, Guoqin Duan, Longyu Sui, Sumera Anwar, Jingyu Ao, Guangdi Liu, Zhenhuai Xu, Caian Zhang, Meijun Qi, Xuejun Li, Meng Zhao, Butian Wang, Yu Ge

**Affiliations:** 1 College of Tropical Crops, Yunnan Agricultural University, Pu’er, Yunnan, China; 2 Yunnan Provincial Key Laboratory of Coffee, Yunnan Agricultural University, Pu’er, Yunnan, China; 3 Department of Botany, Government College Women University Faisalabad, Faisalabad, Pakistan; 4 Institute of Plant Resources, Yunnan University, Kunming, Yunnan, China

**Keywords:** biological control, coffee, coffee quality, endophytes, plant growth promotion

## Abstract

Coffee production is increasingly constrained by climatic variability, unstable yields, major pests and diseases, and growing demand for consistent bean quality. Endophytic microorganisms that colonize internal coffee tissues may contribute to plant growth, stress tolerance, disease and pest suppression, and postharvest quality-related processes. However, current knowledge remains fragmented because confirmed endophytes, rhizosphere microorganisms, phyllosphere taxa, and fermentation-associated microbiota are often discussed together. This review synthesizes coffee endophyte diversity, tissue-specific distribution, colonization routes, host-selection filters, ecological functions, and application prospects, while explicitly separating direct endophyte evidence from coffee-associated and indirect evidence. We show that coffee endophytes are shaped by host genotype, tissue niche, altitude, shade, management system, developmental stage, and microbial source pools, rather than representing a fixed list of ubiquitous taxa. Mechanistically, coffee endophytes may influence nutrient acquisition, phytohormone balance, stress physiology, salicylic acid- and jasmonic acid/ethylene-mediated defense signaling, reactive oxygen species regulation, PR proteins, lignification, antimicrobial metabolites, and multitrophic plant-microbe-insect interactions. Current evidence is strongest for isolation, community description, *in vitro* screening, and short-term greenhouse or pot studies, whereas field persistence, functional stability, biosafety, and formulation remain insufficiently validated. We propose an application-oriented pipeline linking tissue-specific isolation, evidence-level classification, host reinoculation, colonization tracking, multi-omics, synthetic consortia, multi-environment trials, and product development. This synthesis clarifies how coffee endophyte research can move from descriptive microbiome inventories towards reliable microbial tools for sustainable coffee cultivation, integrated pest and disease management, and quality-oriented processing.

## 1 Introduction

The genus *Coffea* comprises perennial evergreen woody plants in the family Rubiaceae. Although the genus includes several species, global commercial production is dominated mainly by *Coffea arabica* L. and *C. canephora Pierre* ex A. Froehner (Davis et al., 2006; Duong et al., 2020). Coffee is a high-value tropical crop whose growth, productivity, and bean quality are strongly influenced by temperature, rainfall, shade conditions, soil properties, and agronomic management practices (Worku, 2023; DaMatta et al., 2025). Climate warming, altered precipitation patterns, and more frequent extreme weather events are expected to shift suitable coffee-growing areas and increase uncertainty in yield and quality (Davis et al., 2012; Bunn et al., 2015; Bilen et al., 2022). At the same time, major biotic stresses, especially coffee leaf rust caused by *Hemileia vastatrix* and the coffee berry borer, *Hypothenemus hampei*, continue to threaten coffee production and may be further influenced by changing climatic conditions (Jaramillo et al., 2011; Talhinhas et al., 2017; Koutouleas et al., 2024). These pressures have increased interest in sustainable management approaches that reduce dependence on chemical inputs and make better use of beneficial microorganisms in coffee production systems (Duong et al., 2020; Gómez et al., 2021; Asad et al., 2023).

Plant endophytes are commonly defined as microorganisms that colonize internal plant tissues during at least part of their life cycle without causing visible disease symptoms. They mainly include bacteria and fungi, although their ecological roles may be beneficial, neutral or context-dependent (Santoyo et al., 2016; Afzal et al., 2019). Endophytes can influence plant growth and health through several mechanisms, including nutrient mobilization, phytohormone regulation, stress mitigation, induced host defense, antimicrobial metabolite production, volatile organic compounds, and cell wall-degrading enzyme activity (Afzal et al., 2019; Compant et al., 2010; Santoyo et al., 2016; Zhang et al., 2025). In coffee, diverse bacterial and fungal endophytes have been isolated or detected from seeds, leaves, roots, stems, cherries, and other tissues (Duong et al., 2021; Fulthorpe et al., 2020; Oliveira et al., 2013; Vega et al., 2008, 2010). Studies on coffee-associated microbiomes and seed microbiomes have further expanded our understanding of microbial sources, tissue specificity, community assembly, and potential ecological functions in coffee plants (Vaughan et al., 2015; Wu et al., 2025). In parallel, research on postharvest fermentation microbiota has shown that microbial communities can influence metabolite transformation and quality formation during coffee processing (De Bruyn et al., 2016; Haile and Kang, 2019; Kutos et al., 2025; Shen et al., 2024). However, fermentation microbiota should be distinguished from confirmed endophytes unless the microbial taxa are traced to internal fruit or seed tissues.

Previous reviews have summarized coffee-associated endophytes, plant growth-promoting microorganisms, and coffee microbiota in relation to crop protection, sustainable management, and processing quality (Duong et al., 2020; Gómez et al., 2021; Asad et al., 2023). However, recent studies on coffee endophyte diversity, tissue-specific community structure, seed and fruit microbiomes, pathogen resistance, and endophyte-based application pathways require a more integrated synthesis (Angamarca et al., 2024; González et al., 2024; Kutos et al., 2025; Obieze et al., 2025; Wu et al., 2025). With the development of plant-microbiome and holobiont concepts, coffee microbial ecology is also shifting from the description of individual strains toward a broader understanding of how host plants, environmental conditions, management systems, and microbial communities interact (Trivedi et al., 2020; Xiong et al., 2021). For a perennial woody crop such as coffee, this perspective is important because microbial communities may vary across tissues, developmental stages, seasons, altitudes, shade systems, and cultivation practices (Fulthorpe et al., 2020; González et al., 2024; Obieze et al., 2025; Vega et al., 2010).

The novelty of this review is not a simple compilation of genera reported from coffee tissues. Many genera recorded as coffee endophytes are widespread in other crops and cannot, by themselves, explain coffee-specific benefits. The contribution of this review is the integration of four elements that are usually treated separately: (i) confirmed coffee endophyte diversity across tissues and ecological gradients; (ii) the distinction between true endophytes, rhizosphere/phyllosphere microorganisms, and fermentation-associated microbiota; (iii) mechanistic interpretation of growth promotion, stress tolerance, host defense, disease suppression, and pest interactions; and (iv) an evidence-based pathway for translating candidate strains into nursery, field, biocontrol, and postharvest applications. This structure allows the review to identify not only which microorganisms have been reported, but also where the evidence is strong, where it is indirect, and which research steps are required before farmer-level use.

## 2 Diversity and community composition of coffee endophytes

### 2.1 Coffee-associated microhabitats and ecological niches

Coffee is mainly cultivated in tropical and subtropical regions, where plant growth, productivity, and microbial communities are influenced by altitude, soil type, slope aspect, intercropping systems, shade level, and management practices (Bilen et al., 2022; Ge et al., 2023; Worku, 2023; Zhang et al., 2023; Zhao et al., 2024). These factors create heterogeneous coffee-associated microhabitats, including bulk soil, rhizosphere, root endosphere, stem tissues, leaves, fruits, seeds, and postharvest processing environments. However, these habitats should not be treated as equivalent. Soil and rhizosphere communities act mainly as external microbial reservoirs, whereas endophytic communities refer to microorganisms that colonize internal plant tissues.

Studies on Yunnan-grown *C. arabica* have shown that slope aspect, altitude, and intercropping can alter rhizospheric soil properties and microbial community structure (Ge et al., 2023; Zhang et al., 2023; Zhao et al., 2024). These studies are useful for understanding the environmental sources from which some endophytes may be recruited, but they do not directly represent internal endophytic communities unless endosphere sampling is included. Therefore, rhizosphere evidence should be used to explain microbial reservoirs and environmental filtering, while endophyte-specific studies should support claims about internal tissue colonization.

Coffee-associated niches show clear compartmentalization. Culture-dependent and sequencing-based studies have reported distinct microbial communities in roots, leaves, fruits, cherries, and seeds (Fulthorpe et al., 2020; Obieze et al., 2025; Oliveira et al., 2013; Vega et al., 2005, 2010). Coffee seeds and cherries are particularly important because they may serve as reservoirs for microbial persistence, seedling establishment, and postharvest microbial succession (Johnston-Monje et al., 2021; Mahatmanto et al., 2023; Tenea et al., 2025; Vaughan et al., 2015). For example, endophytic fungi have been reported from green coffee seeds (Vega et al., 2008), and endophytic microbial diversity has been described in *C. arabica* cherries from southeastern Brazil (Oliveira et al., 2013). These findings indicate that coffee endophytes are not restricted to vegetative tissues but also occur in reproductive and postharvest-relevant tissues.

Postharvest processing environments further reshape coffee-associated microbial communities. Fermentation and processing conditions influence microbial succession, metabolite profiles, and flavor-related outcomes (De Bruyn et al., 2016; Haile and Kang, 2019; Kutos et al., 2025; Shen et al., 2024). These studies are important for understanding coffee quality formation, but they should be interpreted separately from endophyte diversity studies. In this review, postharvest microbiota are considered relevant when they are linked to fruit- or seed-associated microbial reservoirs, but they are not automatically treated as confirmed endophytes.

### 2.2 Community composition and influencing factors of coffee endophytes

Available culture-dependent and molecular evidence indicates that coffee endophytic bacteria are mainly affiliated with Pseudomonadota, Bacillota, and Actinomycetota (Vega et al., 2005; Duong et al., 2021; Lopez et al., 2025). At the genus level, *Pseudomonas*, *Enterobacter*, *Acetobacter*, *Gluconacetobacter*, *Burkholderia*, *Acinetobacter*, *Herbaspirillum*, *Methylobacterium*, *Bacillus*, *Paenibacillus*, and other Actinobacterial genera have been reported from coffee tissues or closely associated coffee habitats (Duong et al., 2021; Jiménez-Salgado et al., 1997; Silva et al., 2012; Vega et al., 2005). Early work identified *Acetobacter diazotrophicus* and other nitrogen-fixing acetobacteria associated with *C. arabica*, supporting the view that coffee plants can harbor diazotrophic bacterial resources (Jiménez-Salgado et al., 1997). Later studies reported culturable coffee bacterial endophytes with functional traits related to growth promotion, pathogen inhibition, and nematode suppression (Shiomi et al., 2006; Silva et al., 2012; Duong et al., 2021).

Coffee-associated fungal endophytes are mainly affiliated with Ascomycota, with some Basidiomycota also reported (Oliveira et al., 2013, 2014; González et al., 2024; Vega et al., 2010). Commonly reported genera include *Colletotrichum*, *Xylaria*, *Fusarium*, *Penicillium*, *Trichoderma*, *Cladosporium*, *Aspergillus*, *Diaporthe*, *Pestalotiopsis*, *Phoma/Phomopsis*, *Muscodor/Induratia*, and *Beauveria* (Peterson et al., 2005; Posada et al., 2007; Vega et al., 2010; Mulaw et al., 2013; Li et al., 2022; Angamarca et al., 2024). These fungi have been isolated or detected from leaves, roots, stems, seeds, berries, and cherries, although the dominant taxa vary with tissue type, coffee genotype, geographic origin, management system, and detection method (Fulthorpe et al., 2020; González et al., 2024; Obieze et al., 2025; Oliveira et al., 2013; Vega et al., 2010).

Community composition also differs across tissues and ecological gradients. Root endophytes of *C. arabica* vary across climatic gradients and are linked with host functional traits (Fulthorpe et al., 2020). Foliar fungal endophytes differ among coffee varieties, agroecosystems, and management systems (González et al., 2024; Oliveira et al., 2014; Saucedo-García et al., 2014). Recent endosphere-level evidence further shows that altitude and shade influence bacterial and fungal communities in leaves and fruits of *C. arabica* (Obieze et al., 2025). Therefore, coffee endophyte diversity should be interpreted as tissue-specific and context-dependent, rather than as a fixed list of taxa.

Several genera reported as coffee endophytes, including *Colletotrichum*, *Fusarium*, *Aspergillus*, and *Penicillium*, may include beneficial, neutral, opportunistic, or pathogenic members depending on strain identity, host condition, and environmental context. Therefore, their presence in asymptomatic coffee tissues should not automatically be interpreted as beneficial. Strain-level identification, functional validation, and biosafety evaluation are needed before such taxa are considered for application. The evidence categories used to interpret coffee endophytes and coffee-associated microorganisms are summarized in **Table 1**.

**Table 1:** Evidence categories used to classify coffee endophytes and coffee-associated microorganisms.

Evidence category	Typical tissue/niche	Representative studies	Main interpretation	Key limitation
Confirmed coffee endophyte	Surface-sterilized roots, leaves, stems, seeds	Vega et al. (2005, 2008, 2010); Shiomi et al. (2006); Silva et al. (2012); Duong et al. (2021)	Direct evidence of internal coffee-associated microorganisms	Functional effects often need reinoculation and field validation
Coffee-associated rhizosphere isolate	Rhizosphere, rhizoplane, root washing solution	Muleta et al. (2013); Teshome et al. (2017); Kunwar et al. (2018)	Useful reservoir for nutrient-mobilizing microbes	Not confirmed as endophytes unless internal colonization is shown
Coffee-associated phyllosphere/lesion isolate	Rust lesions, diseased leaves, leaf surface-associated material	Luiz et al. (2024); Wulansari et al. (2023)	Useful for local biocontrol discovery	Should not be treated as true endophytes without colonization proof
Fruit/seed-associated microbiota	Seeds, cherries, whole fruits	Vega et al. (2008); Oliveira et al. (2013); Mahatmanto et al. (2023); Kutos et al. (2025)	May contribute to reproductive-tissue microbiomes and fermentation inocula	Often does not separate surface and internal microbiota
Fermentation-associated microbiota	Fermentation mass, slurry, processing environment	Pereira et al. (2022); Todhanakasem et al. (2024); Tenea et al. (2025)	Important for quality, aroma, and metabolite formation	Not equivalent to confirmed endophytes
Indirect coffee-derived functional evidence	Coffee-derived strains tested in other hosts or biochemical assays	Jirakkakul et al. (2023); Numponsak et al. (2018)	Provides mechanistic clues	Coffee plant-level validation is still lacking

### 2.3 Context-dependent variation in coffee endophyte communities

A synthesis of coffee endophyte diversity should move beyond genus-level lists because many genera reported from coffee, such as *Pseudomonas*, *Bacillus*, *Enterobacter*, *Trichoderma*, *Colletotrichum*, *Fusarium*, *Penicillium*, and *Beauveria*, are also common in many other plant species (Afzal et al., 2019; Compant et al., 2010; Santoyo et al., 2016). Their occurrence becomes meaningful only when interpreted in relation to host tissue, coffee species or variety, geography, management system, plant age, and functional validation. Available coffee studies indicate that root, leaf, fruit, and seed tissues act as distinct filters for microbial colonization, while altitude, shade, climate, and agricultural practices further alter community composition (Vega et al., 2010; Fulthorpe et al., 2020; González et al., 2024; Obieze et al., 2025). For example, foliar endophyte communities may differ between coffee varieties and management systems, root endophytes may vary across climatic gradients and host functional traits, and fruit/seed microbiomes may shift with ripening stage and processing context (González et al., 2024; Mahatmanto et al., 2023; Tenea et al., 2025). Therefore, the most useful conclusion is not that coffee contains a large number of microbial genera, but that coffee plants assemble context-dependent endophytic communities through repeated filtering by genotype, tissue niche, environment, and management.

This interpretation has direct application value. For seedling-vigor applications, root-derived strains with stable colonization and nutrient- or drought-related traits should be prioritized (Duong et al., 2021; Jasso-Arreola et al., 2025; Pratiwi et al., 2020). If the target is coffee leaf rust, leaf- or stem-associated endophytes that interfere with *Hemileia vastatrix* or activate foliar defenses are more relevant. If the target is postharvest quality, fruit- and seed-associated microorganisms should be evaluated through controlled fermentation, metabolomics, and sensory validation (Kutos et al., 2025; Pereira et al., 2022; Todhanakasem et al., 2024). Thus, endophyte selection should be target-driven rather than based only on taxonomic occurrence.

## 3 Sources, colonization, and community assembly of coffee endophytes

**Figure 1:**
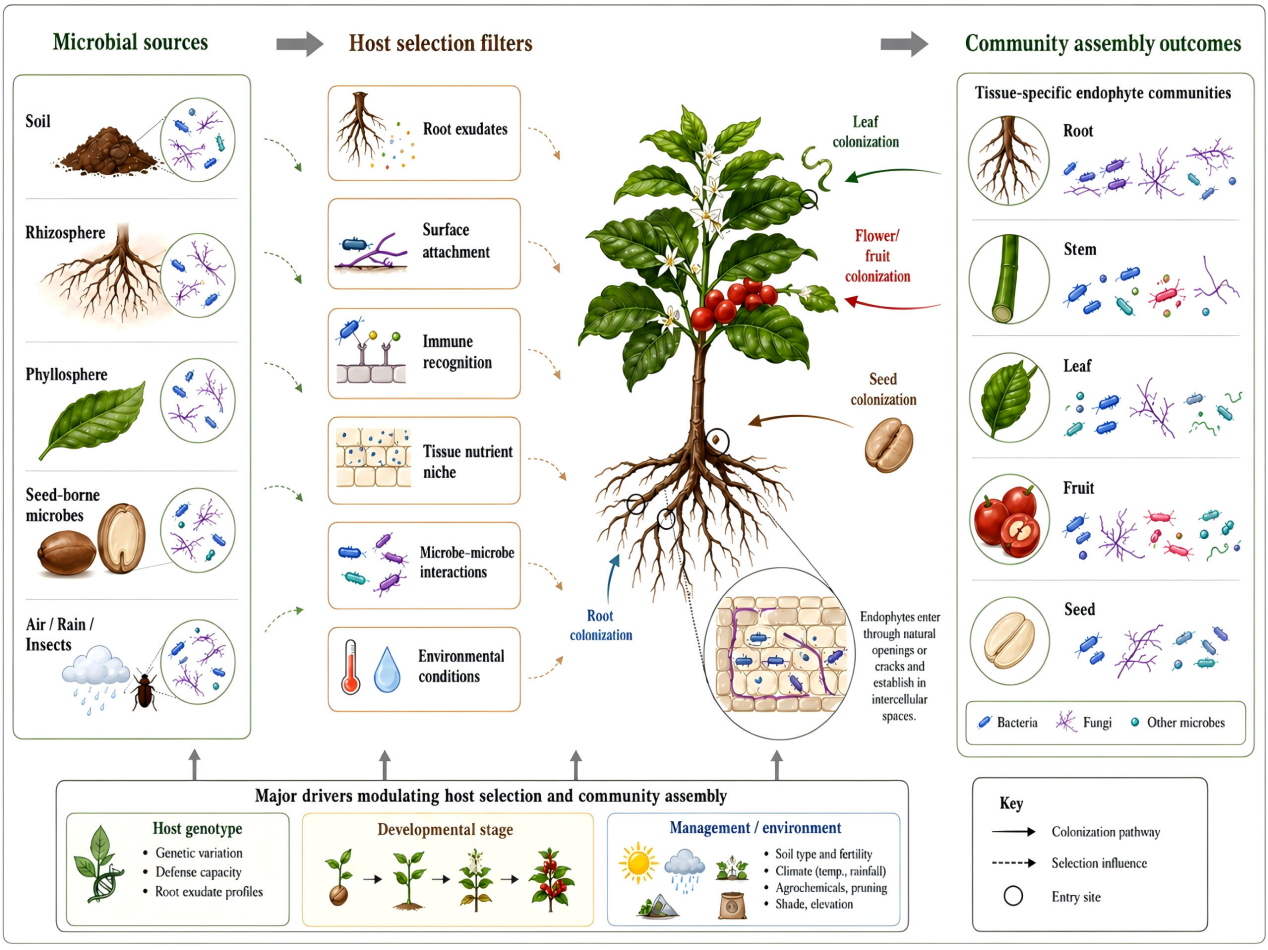
Conceptual framework of the sources, host filtering, colonization pathways, and community assembly outcomes of coffee endophytes.

Coffee endophytes originate from multiple microbial reservoirs, including soil, the rhizosphere, plant surfaces, seeds, fruits, airborne particles, rain splash, and, in some cases, insect- or arthropod-mediated dispersal. However, the relative contribution of each source may differ among bacterial and fungal endophytes, plant tissues, developmental stages, and production environments. In coffee, available evidence supports both horizontal acquisition from external habitats and seed-associated transmission as important pathways contributing to the establishment of internal microbial communities. A conceptual framework showing the sources, host filtering, colonization pathways, and community assembly outcomes of coffee endophytes is presented in **Figure 1**.

### 3.1 Sources, transmission routes, and colonization pathways of endophytes

The rhizosphere is widely considered one of the major entry points for plant-associated bacteria into internal tissues. Root exudates contain sugars, amino acids, organic acids, phenolics, and other compounds that can support microbial growth and act as chemical signals for chemotaxis, attachment, and early colonization. After reaching the root surface, microorganisms may enter plant tissues through root hairs, lateral-root emergence sites, cracks in the epidermis, natural openings, or wounds. Some fungal endophytes can also penetrate plant surfaces through hyphal growth, appressoria-like structures, or enzymatic softening of the epidermal barrier. These colonization pathways are well established in general plant endophyte studies, although direct mechanistic evidence in coffee remains comparatively limited (Rosenblueth and Martínez-Romero, 2006; Compant et al., 2010; Santoyo et al., 2016; Afzal et al., 2019).

In addition to horizontal acquisition from soil, rhizosphere, and plant surfaces, seed-associated microorganisms can contribute to the early assembly of plant microbiomes (Truyens et al., 2015; Li et al., 2023). Johnston-Monje et al. (2021) showed that seedlings of several plant species, including *C. arabica* var. Geisha, could establish rhizosphere, root endophytic, and phyllosphere microbial communities when seeds were the only microbial source. This finding supports the view that seed-transmitted bacteria and fungi can contribute to the initial microbiome of young plants. However, seed transmission should not be assumed to be universal for all coffee endophytes; rather, it should be treated as one important source that interacts with later environmental acquisition.

Coffee-specific studies also support the importance of seeds as endophyte habitats. Vega et al. (2005) isolated diverse bacterial endophytes from different *C. arabica* tissues and reported that seeds yielded a high number of bacterial isolates. Vega et al. (2008) further documented fungal endophytes in green coffee seeds from different origins, indicating that coffee seeds can harbor diverse internal fungi. These findings suggest that seed-associated endophytes may contribute to the early microbial reservoir of coffee plants and may also be relevant to seed microbiome persistence and postharvest microbial dynamics. Vaughan et al. (2015) further emphasized the coffee seed microbiome as an important target for understanding microbial sources, persistence, and potential links between seed-associated microbes and coffee processing.

Overall, coffee endophyte colonization can be understood as a stepwise process involving microbial sourcing, surface arrival, attachment, entry into plant tissues, survival inside host microenvironments, and local persistence or redistribution within tissues. Entry into plant tissue, however, does not necessarily indicate stable colonization. Long-term persistence depends on host selection, immune tolerance, tissue chemistry, microbial competitiveness, and compatibility with the resident microbiome. Therefore, future studies should distinguish between transient internal detection and stable endophytic colonization, especially when evaluating candidate strains for application.

### 3.2 Host selection and community assembly

Coffee endophytic communities are not formed simply by random entry of external microorganisms into plant tissues. Instead, their assembly is shaped by the combined effects of external microbial reservoirs, host-mediated filtering, tissue-specific microenvironments, and environmental conditions. General plant microbiome studies show that plants influence microbial recruitment and persistence through root exudates, surface traits, immune recognition, nutrient availability, and tissue-specific physicochemical conditions (Compant et al., 2010; Trivedi et al., 2020; Xiong et al., 2021). These mechanisms provide a useful framework for interpreting coffee endophyte assembly, but coffee-specific evidence should be considered separately from general plant-microbiome theory.

Several coffee studies support tissue-specific patterns in endophytic and associated microbial communities. Fungal endophytes have been reported from green coffee seeds (Vega et al., 2008), foliar endophytic communities vary with coffee variety and agricultural management (González et al., 2024), and root endophytes differ across climatic gradients and host functional traits (Fulthorpe et al., 2020). Endophytic microbial diversity has also been reported in coffee cherries (Oliveira et al., 2013), while coffee seed microbiome studies suggest that seeds may act as reservoirs for microorganisms relevant to plant development and postharvest processes (Vaughan et al., 2015). These findings indicate that roots, leaves, fruits, and seeds provide distinct ecological niches for microbial colonization and persistence.

The soil–rhizosphere–root continuum is also important for coffee microbiome assembly, but rhizosphere studies should be distinguished from true endophyte studies. Research on coffee rhizospheric soil has shown that slope aspect, intercropping, altitude, and soil chemical properties can influence microbial community structure (Ge et al., 2023; Zhang et al., 2023; Zhao et al., 2024). These studies help explain the environmental reservoirs from which endophytes may be recruited, but they do not directly demonstrate internal colonization unless endosphere sampling or colonization validation is included. Therefore, rhizosphere evidence should be used to support external microbial sourcing and environmental filtering, while endophyte-specific studies should support claims about internal tissue communities.

Recent endosphere-level work has further strengthened the coffee-specific evidence. Obieze et al. (2025) investigated bacterial and fungal endosphere communities in leaves and fruits of *C. arabica* grown in montane agroforestry systems in Mozambique. Their study showed that altitude was a major driver of endophytic microbial community structure, while shade also influenced bacterial and fungal community composition. This indicates that aboveground coffee endophytes are shaped not only by tissue niche, but also by environmental gradients and management context.

Taken together, the assembly of coffee endophytic communities can be conceptualized as a continuous process involving microbial sourcing from seeds, soil, rhizosphere, phyllosphere, fruits, and surrounding environments; host filtering during entry and persistence; and niche differentiation within roots, leaves, fruits, and seeds. This framework helps move coffee endophyte research beyond simple taxonomic description toward a more integrated understanding of community assembly, tissue specificity, environmental filtering, and functional stability.

### 3.3 Coffee-specific selection filters and their manipulation in production systems

Coffee endophyte assembly is likely governed by general plant microbiome principles, but coffee has several crop-specific features that should be considered when interpreting host selection. Coffee is a perennial woody crop with long-lived roots, persistent leaves, repeated flowering and fruiting cycles, and strong exposure to altitude, shade, pruning, rainfall, soil fertility, pest pressure, and postharvest handling. These features create stable but heterogeneous microhabitats where microorganisms must tolerate host immune responses, tissue chemistry, nutrient availability, and competition with resident microbiota. Therefore, coffee endophyte assembly should not be viewed as random microbial entry into plant tissues but as repeated filtering by genotype, tissue niche, environment, management, and developmental stage.

Coffee tissues may impose particularly important chemical and nutritional filters. Roots release exudates that influence microbial recruitment from soil and rhizosphere reservoirs, whereas leaves, stems, fruits, and seeds differ in nutrient availability, surface traits, defense status, and opportunities for microbial entry. Coffee tissues also contain biologically active metabolites, including caffeine, chlorogenic acids, phenolics, sugars, and fruit mucilage components, which may affect microbial survival, persistence, or functional expression. These filters may partly explain why roots, leaves, fruits, and seeds harbor different microbial assemblages and why endophyte communities vary across coffee-growing regions, agroforestry systems, shade levels, and management practices (Vega et al., 2010; Fulthorpe et al., 2020; González et al., 2024; Obieze et al., 2025). However, most current studies remain descriptive, and direct experiments linking individual coffee metabolites or host traits with endophyte selection are still limited.

The practical value of these filters is that they provide entry points for microbiome management. In nurseries, seed treatment, substrate management, and seedling inoculation can be used to introduce candidate endophytes before transplanting, when colonization can be monitored more easily. For seedling vigor, root-derived strains with stable colonization and traits related to nutrient acquisition, auxin production, ACC deaminase activity, or drought tolerance should be prioritized over strains selected only on the basis of taxonomic identity. In the field, shade management, organic amendments, intercropping, reduced pesticide disturbance, pruning practices, and soil fertility management may influence microbial reservoirs and increase the probability of beneficial recruitment from soil, rhizosphere, phyllosphere, and litter-associated sources. These practices should be viewed as indirect but realistic tools for shaping the microbial pool from which coffee endophytes are recruited.

For disease and pest management, coffee-specific filtering also has practical implications. Leaf-, stem-, or root-associated endophytes isolated from healthy plants, resistant varieties, or disease-suppressive farms may be more relevant than strains obtained from unrelated hosts. Candidate strains should be screened not only for antagonism against pathogens or pests, but also for their ability to colonize the target coffee tissue, persist under field conditions, and remain compatible with existing management practices. For coffee leaf rust, foliar or stem-associated endophytes may be more directly relevant than rhizosphere isolates, whereas for root health and nematode suppression, root-derived strains are more appropriate. This target-driven selection strategy can improve the practical relevance of endophyte-based applications.

For fruit quality and postharvest management, fruit- and seed-associated microbiota should be interpreted separately from confirmed vegetative endophytes. Coffee cherry maturity, processing method, fermentation environment, water source, equipment hygiene, and local microbial reservoirs may all influence the microorganisms entering fermentation. Studies on cherry and fermentation microbiota indicate that coffee variety, ripeness stage, shade management, farm environment, and fermentation conditions can shape microbial communities and quality-related metabolites (Pereira et al., 2022; Mahatmanto et al., 2023; Todhanakasem et al., 2024; Kutos et al., 2025; Tenea et al., 2025). However, fermentation-associated microorganisms should not be treated as confirmed endophytes unless their internal fruit or seed origin is demonstrated.

Overall, coffee host filtering should be interpreted as both an ecological process and an applied management opportunity. The most useful approach is not simply to list microbial genera found in coffee, but to identify which microorganisms are selected by specific coffee tissues, genotypes, environments, and production systems, and then test whether these microorganisms provide stable benefits under realistic nursery, field, or postharvest conditions. Future work should combine colonization tracking, host genotype comparison, tissue chemistry, soil and shade management data, multi-omics analysis, and plant performance measurements to determine which filters can be manipulated reliably for coffee production.

### 3.4 Interactive responses to climate change and environmental stresses

Climate change is expected to alter the ecological conditions required for coffee growth. Rising temperatures, altered rainfall patterns, drought events, and shifting agroclimatic suitability can affect coffee distribution, productivity, and production stability (Davis et al., 2012; Bunn et al., 2015; Bilen et al., 2022; DaMatta et al., 2025). Climate change may also intensify or redistribute important coffee pests and diseases, including coffee leaf rust caused by *Hemileia vastatrix* and the coffee berry borer, *Hypothenemus hampei* (Jaramillo et al., 2011; Talhinhas et al., 2017; Castillo et al., 2020; Koutouleas et al., 2024). These changes are likely to affect not only coffee plants directly, but also the microbial reservoirs and plant–microbe interactions that contribute to endophyte recruitment, persistence, and function.

For coffee endophytes, the effects of climate change should not be understood only as shifts in microbial relative abundance. Environmental stress can modify host physiology, root exudation, tissue nutrient status, immune regulation, phenology, disease pressure, and pest interactions. These changes may alter which microorganisms are recruited from the rhizosphere or plant surface, which taxa can persist inside tissues, and which microbial functions are expressed under stress. However, direct studies linking climate-change variables with coffee endophyte community assembly and functional expression remain limited. Current interpretation therefore relies partly on coffee ecological studies, coffee microbiome studies, and broader plant-microbiome theory (Duong et al., 2020; Trivedi et al., 2020; Xiong et al., 2021; Asad et al., 2023).

Under drought, heat, nutrient limitation, or combined stresses, plant endophytes and plant growth-promoting microorganisms may support host adaptation by regulating phytohormone balance, improving root growth, enhancing nutrient acquisition, producing osmoprotective or antioxidant-related effects, and modulating defense responses (Glick, 2014; Backer et al., 2018; Lopez et al., 2025). These effects may involve modulation of ABA/ethylene balance, reduction of stress-induced ROS damage through antioxidant enzymes, improved osmotic adjustment, maintenance of root hydraulic function, and better acquisition of nutrients under drought or heat stress. Among these mechanisms, ACC deaminase-producing bacteria are particularly important because they can lower stress-induced ethylene levels and thereby reduce stress-related growth inhibition (Glick, 2014). In coffee, Jasso-Arreola et al. (2025) reported that *Pantoea* sp. RCa62, an ACC deaminase-producing bacterium isolated from the rhizosphere of *C. arabica*, improved leaf area, relative water content, biomass accumulation, and root development under pot-based drought stress. This study provides useful evidence for coffee-associated rhizobacteria in drought mitigation, but it should not be presented as direct endophyte evidence unless internal colonization is confirmed.

Therefore, climate-stress discussion in coffee endophyte research should clearly distinguish three evidence levels: direct evidence from coffee endophytes, evidence from coffee-associated rhizosphere or phyllosphere microorganisms, and mechanistic inference from general plant microbiome studies. Future work should focus on identifying stress-responsive core endophytes, confirming colonization and persistence under stress, and linking microbial shifts with measurable coffee phenotypes. Long-term field monitoring, controlled drought or heat experiments, strain reinoculation, multi-omics analysis, and host physiological assessment will be necessary to clarify how coffee endophytes contribute to stress adaptation under realistic production conditions.

## 4 Main ecological functions of coffee endophytes

### 4.1 Nutrient mobilization and growth-promoting mechanisms

Mineral nutrients, particularly nitrogen, phosphorus, potassium, and iron, are essential for coffee growth, seedling establishment, and bean quality formation. Coffee-associated microorganisms have been reported to possess several plant growth-promoting traits, including nitrogen fixation, phosphate solubilization, siderophore production, indole-3-acetic acid production, ACC deaminase activity, and extracellular enzyme activities (Duong et al., 2021; Jasso-Arreola et al., 2025; Muleta et al., 2013; Pratiwi et al., 2020; Silva et al., 2012). However, these reports differ substantially in their evidence strength.

Confirmed coffee endophytes provide direct evidence when isolates are recovered from surface-sterilized internal tissues and show plant growth-promoting traits, reinoculation capacity, colonization ability, or plant performance effects (Duong et al., 2021; Pratiwi et al., 2020; Silva et al., 2012). Coffee-associated rhizosphere isolates provide useful but indirect evidence because they may improve nutrient availability around roots without necessarily entering internal tissues (Jasso-Arreola et al., 2025; Muleta et al., 2013; Teshome et al., 2017). Coffee-derived strains tested in non-coffee hosts provide mechanistic clues, but they should not be treated as validated coffee bioinoculants unless their effects are confirmed in coffee plants (Jirakkakul et al., 2023).

Among confirmed coffee endophytes, reported growth-promoting mechanisms include IAA production, phosphate-solubilization traits, siderophore production, extracellular enzyme activity, and antagonistic traits that may indirectly support plant growth by reducing pathogen pressure (Duong et al., 2021; Numponsak et al., 2018; Pratiwi et al., 2020; Silva et al., 2012). Auxin-producing root endophytes may influence root branching, root hair development, and absorptive surface area, thereby improving nutrient and water acquisition. Siderophore-producing bacteria may enhance iron acquisition under iron-limited conditions and may also restrict pathogen growth by competing for available iron in the endosphere. Extracellular enzymes, including chitinases, lipases, esterases, gelatinases, and related hydrolytic enzymes, may contribute to nutrient transformation or pathogen suppression, although their activity inside coffee tissues remains insufficiently verified.

Early work reported nitrogen-fixing *Acetobacter* groups from coffee plant tissues and rhizospheric soils, suggesting that coffee plants can harbor diazotrophic microorganisms in both internal and root-associated niches (Jiménez-Salgado et al., 1997). This finding supports the potential presence of nitrogen-related microbial functions in coffee systems. However, the quantitative contribution of these diazotrophic microorganisms to coffee nitrogen nutrition remains insufficiently demonstrated. Future studies should therefore combine nitrogen-fixation assays with colonization tracking, isotopic nitrogen approaches, plant nitrogen analysis, and biomass or yield measurements before strong claims are made about endophyte-mediated nitrogen nutrition in coffee.

For phosphorus mobilization, much of the available evidence comes from rhizosphere bacteria rather than confirmed endophytes. Phosphate-solubilizing rhizobacteria isolated from the rhizosphere of *C. arabica* have been shown to possess phosphorus-solubilizing capacity and to support seedling growth under controlled conditions (Teshome et al., 2017; Kunwar et al., 2018). These studies are valuable for identifying nutrient-mobilizing microbial resources around coffee roots, but they should not be interpreted as direct evidence of endophyte-mediated phosphorus mobilization unless internal colonization and plant-level phosphorus responses are demonstrated. Therefore, phosphate solubilization should be discussed as a promising trait shared by coffee-associated microorganisms, with direct endophytic relevance requiring further validation.

Among direct coffee endophyte studies, Pratiwi et al. (2020) isolated IAA-producing bacterial strains from the roots of *C. canephora* and *C. arabica*. The strongest IAA-producing isolates, SS.E2 from *C. canephora* and SW.E9 from *C. arabica*, were identified as *Bacillus cereus* and produced 110.73 and 257.16 μg mL^-^¹ IAA, respectively. These findings indicate that coffee root endophytes may contribute to auxin-mediated growth regulation. However, because *B. cereus* includes strains with biosafety concerns, strain-level safety evaluation, genome-based screening, and pathogenicity testing are necessary before any application-oriented use.

Duong et al. (2021) further showed that culturable endophytic bacteria isolated from coffee roots and seeds in Vietnam expressed multiple plant growth-promoting traits, including phosphate solubilization, indole compound production, siderophore production, hydrogen cyanide production, and extracellular enzyme activities such as esterase, lipase, gelatinase, and chitinase. This study provides direct evidence that coffee endophytic bacteria can carry multiple functional traits relevant to nutrient mobilization, hormone-related growth regulation, iron acquisition, and microbial antagonism. Nevertheless, *in vitro* functional traits should be followed by reinoculation, colonization confirmation, and plant-performance validation to determine whether these traits operate effectively inside coffee plants.

Genomic and metabolic studies provide additional insight into the growth-promoting potential of coffee-derived endophytes. *Methylobacterium* sp. NMS14P, isolated from coffee roots, carries genes associated with phytohormone production, ACC deaminase activity, and cytokinin-related metabolism (Jirakkakul et al., 2023). However, because its plant growth-promoting effects were mainly tested in maize, chili, and sugarcane rather than coffee, this evidence should be treated as indirect functional evidence rather than confirmed coffee growth-promotion evidence. Similarly, the endophytic fungus *Colletotrichum fructicola* CMU-A109, isolated from *C. arabica*, was shown to produce IAA through the indole-3-acetamide and tryptophan 2-monooxygenase pathways (Numponsak et al., 2018). This provides useful mechanistic evidence that coffee-derived fungal endophytes may contribute to hormone-related growth regulation, although direct effects on coffee growth remain to be confirmed.

**Figure 2:**
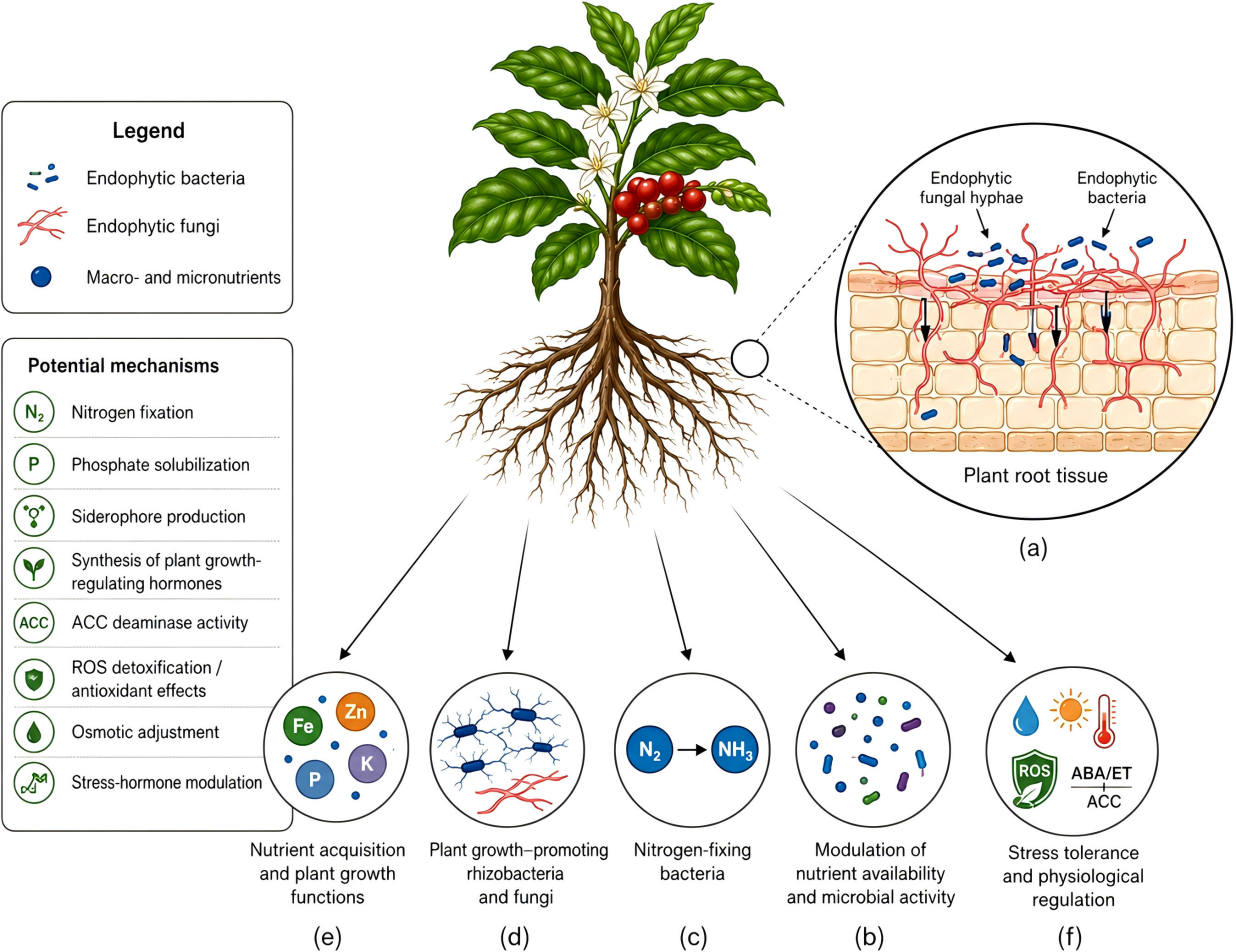
Potential mechanisms by which endophytes and rhizosphere microorganisms promote nutrient acquisition, plant growth, and stress tolerance in coffee. **(a)** Endophytic fungal hyphae and bacteria colonize internal root tissues; **(b)** microbial communities modulate nutrient availability and microbial activity in the rhizosphere and endosphere; **(c)** nitrogen-fixing bacteria convert atmospheric nitrogen into plant-available nitrogen forms, such as ammonium; **(d)** plant growth-promoting rhizobacteria and fungi interact with coffee roots and contribute to growth-promoting functions; **(e)** microorganisms enhance nutrient acquisition and plant growth through macro- and micronutrient mobilization; **(f)** endophytes and associated microorganisms may contribute to stress tolerance and physiological regulation through ACC deaminase activity, ABA/ethylene modulation, ROS detoxification, antioxidant effects, osmotic adjustment, and maintenance of root function under drought or heat stress.

Overall, available evidence suggests that coffee endophytes and closely associated rhizosphere microorganisms may support plant growth through nutrient mobilization, phytohormone production, siderophore-mediated iron acquisition, ACC deaminase activity, and enzymatic functions. However, confirmed coffee endophytes, rhizosphere isolates, and coffee-derived strains tested in non-coffee hosts should be clearly distinguished (**Table 2**). Potential mechanisms by which endophytes and rhizosphere microorganisms promote nutrient acquisition and plant growth in coffee are summarized in **Figure 2**.

**Table 2:** Coffee endophytes and associated microorganisms involved in nutrient mobilization and plant growth promotion.

Functional category	Microbial taxon/ strain	Source/ tissue	Coffee species/ material	Proposed mechanism	Experimental evidence	Main outcome	Evidence strength	Reference
Nitrogen fixation	Diazotrophic acetobacteria (Acetobacter group)	Internal tissues and rhizosphere	C. arabica plants	Biological nitrogen fixation	Isolation and functional screening	Demonstrated diazotrophic bacteria in coffee-associated niches	Mixed evidence: endophyte + rhizosphere	Jiménez-Salgado et al., 1997
Multifunctional growth promotion	Endophytic bacterial isolates	Roots and seeds	Coffee plants from Vietnam	P solubilization, indole compounds, siderophores, HCN, and hydrolytic enzymes	Culture-based isolation and in vitro PGP screening	80 isolates showed multiple growth-promoting traits	Direct coffee endophyte evidence; functional screening	Duong et al., 2021
Phosphate solubilization	Phosphate-solubilizing rhizobacteria	Rhizosphere	C. arabica	Phosphorus solubilization	Isolation and biochemical screening	Identified phosphate-solubilizing bacteria associated with coffee roots	Rhizosphere evidence, not confirmed as endophyte	Teshome et al., 2017
Seedling growth promotion	Phosphate-solubilizing bacteria	Rhizosphere	Coffee seedlings	Phosphate solubilization and growth stimulation	Seed germination and seedling bioassay	Enhanced seed germination and seedling growth	Rhizosphere evidence; seedling level bioassay	Kunwar et al., 2018
Auxin-mediated growth promotion	Bacillus cereus SS.E2 and SW.E9	Root endosphere	C. canephora and C. arabica	IAA production	Isolation and in vitro IAA assay	Root endophytic isolates showed higher IAA production	Direct coffee endophyte evidence; in vitro biochemical assay; biosafety caution needed	Pratiwi et al., 2020
Multifunctional plant growth promotion	Methylobacterium sp. NMS14P	Root-derived isolate	C. arabica var. Chiang Mai 80 / organic coffee root, Thailand	P solubilization, phosphatase and urease activity, ACC deaminase activity, IAA production, cytokinin-related metabolism, and stress-adaptation traits	Genome sequencing, comparative genomics, in vitro PGP assays, and plant growth tests in non-coffee hosts	NMS14P carried multiple PGP-related genes and traits and promoted growth in maize, chili, and sugarcane	Indirect coffee-derived evidence; coffee growth response and recolonization not validated	Jirakkakul et al., 2023
Auxin/phytohormone production	Colletotrichum fructicola CMU-A109	Leaf endophyte	C. arabica / northern Thailand	IAA biosynthesis via the indole-3-acetamide pathway	Endophyte isolation, multilocus identification, IAA detection, pathway analysis, and coleoptile elongation assay	CMU-A109 produced high IAA levels and stimulated coleoptile elongation	Direct coffee-derived fungal endophyte evidence; biochemical/physiological assay; direct coffee growth effect not validated	Numponsak et al., 2018

### 4.2 Disease and pest control by coffee endophytes

#### 4.2.1 Suppression of fungal diseases

Coffee leaf rust, mainly caused by *Hemileia vastatrix*, remains one of the most destructive fungal diseases limiting coffee productivity and stability (Talhinhas et al., 2017; Castillo et al., 2020; Koutouleas et al., 2024). Other fungal diseases, including coffee tracheomycosis, also threaten coffee health in specific production regions (Mulaw et al., 2013). Endophyte-mediated suppression of coffee diseases should be interpreted through two complementary mechanisms: direct pathogen antagonism and host-mediated resistance. Direct antagonism may include antibiosis, competition for nutrients and infection sites, secretion of lytic enzymes, mycoparasitism, siderophore-mediated iron competition, and production of volatile or diffusible antimicrobial metabolites (Afzal et al., 2019; Compant et al., 2010; Santoyo et al., 2016). These mechanisms are particularly relevant when coffee-derived endophytes suppress *H. vastatrix*, coffee tracheomycosis pathogens, or other fungal pathogens *in vitro*, in detached-leaf assays, or in planta (Angamarca et al., 2024; Mulaw et al., 2013; Pereira et al., 2024; Shiomi et al., 2006).

Host-mediated resistance requires a deeper mechanistic explanation. After internal colonization, microbial elicitors may be recognized by plant pattern-recognition systems, thereby triggering immune signaling and priming of defense responses. In coffee, this model is especially relevant to coffee leaf rust because *H. vastatrix* is a biotrophic pathogen and host resistance is strongly linked to early recognition and defense activation (Koutouleas et al., 2024; Talhinhas et al., 2017). Endophytes may contribute to salicylic acid-associated systemic acquired resistance, jasmonic acid/ethylene-associated induced systemic resistance, reactive oxygen species burst regulation, antioxidant balance, and activation of defense-related genes. Downstream responses may include pathogenesis-related proteins, chitinases, *β*-1,3-glucanases, phenylpropanoid metabolism, lignification, phytoalexin or antimicrobial metabolite accumulation, and cell-wall strengthening. These responses may reduce uredospore germination, restrict haustorium establishment, delay lesion development, or reduce sporulation.

**Figure 3:**
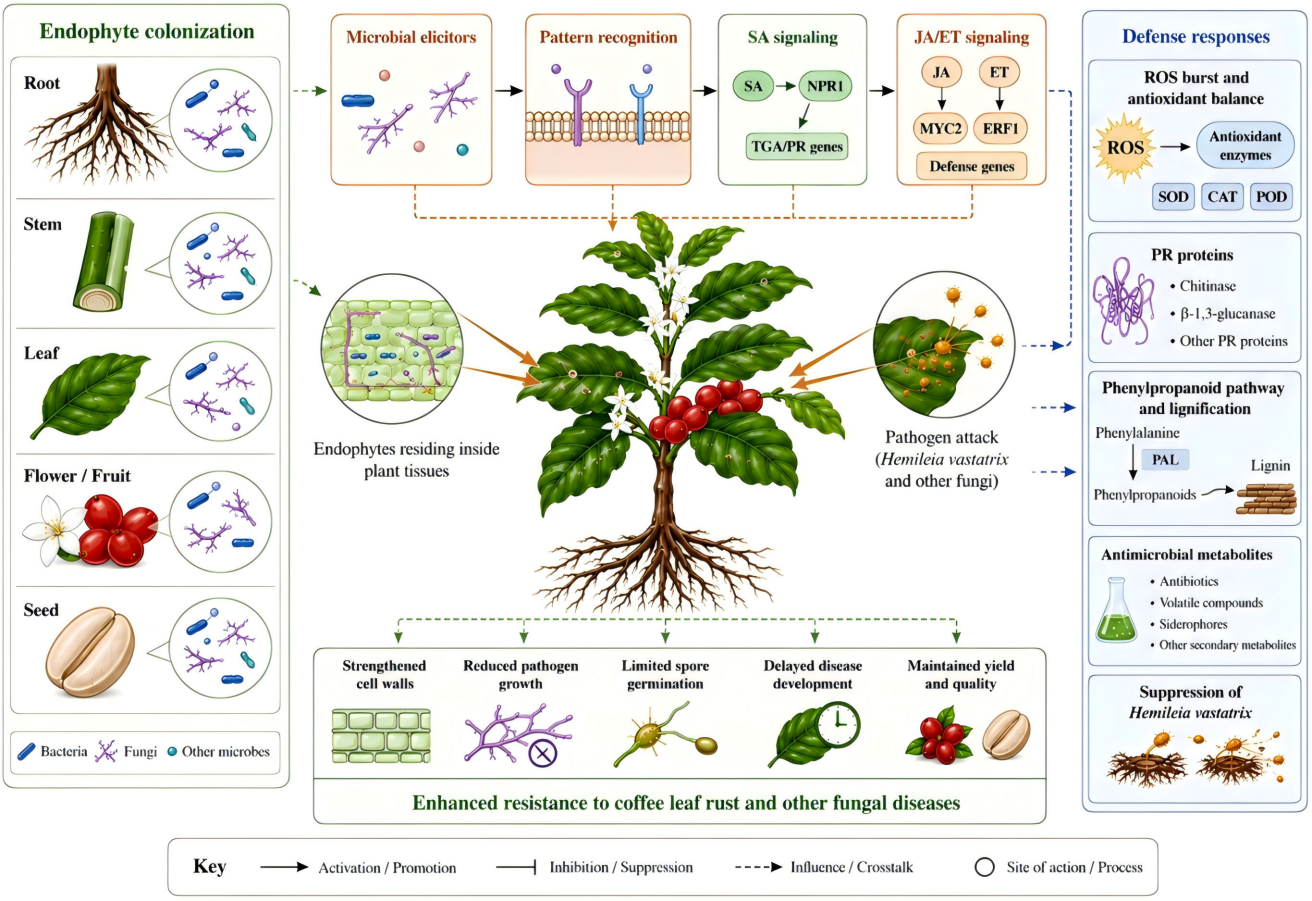
Proposed biochemical and molecular mechanisms of endophyte-mediated defense and disease suppression in coffee. Coffee endophytes may colonize roots, stems, leaves, flowers/fruits, and seeds. After colonization, microbial elicitors may activate host immune signaling, including SA-mediated NPR1/TGA/PR responses and JA/ET-mediated MYC2/ERF1 defense pathways. These responses may regulate ROS burst and antioxidant balance, PR proteins such as chitinases and beta-1,3-glucanases, phenylpropanoid metabolism, lignification, and antimicrobial metabolites. Collectively, these processes may strengthen cell walls, restrict pathogen growth, limit spore germination, delay disease development, and suppress *Hemileia vastatrix* and other fungal pathogens. The proposed mechanisms require further coffee-specific molecular and field validation.

However, the available evidence should be interpreted according to the source and validation level of each microbial candidate. Confirmed endophytes, lesion-associated fungi, phyllosphere antagonists, and rhizosphere microorganisms do not represent the same category of biological control agents (Li et al., 2022; Luiz et al., 2024; Wulansari et al., 2023). Moreover, many of the proposed defense pathways are inferred from coffee–pathogen studies and broader plant–endophyte research and still require coffee-specific molecular validation using colonization tracking, defense-gene expression, enzyme assays, metabolite profiling, and disease-severity assessment. A proposed model of endophyte-mediated defense and disease suppression in coffee is shown in **Figure 3**.

Coffee endophytic bacteria have also been evaluated for the suppression of coffee leaf rust. Shiomi et al. (2006) bioprospected endophytic bacteria from coffee tissues and assessed their potential against *H. vastatrix*. This type of study is important because it directly links coffee-derived endophytic bacteria with disease-control potential, rather than relying only on general plant endophyte mechanisms. In addition, root-derived endophytic fungi may contribute to the suppression of other coffee fungal diseases. Mulaw et al. (2013) reported novel endophytic *Trichoderma* spp. isolated from healthy *C. arabica* roots with the ability to control coffee tracheomycosis. This broadens the relevance of coffee endophytes beyond foliar rust suppression and supports the potential use of tissue-specific endophytes against different fungal pathogens.

Some studies have focused on fungi associated with diseased leaves or rust lesions rather than confirmed internal endophytes. Luiz et al. (2024) isolated fungi from coffee leaves affected by leaf rust and identified taxa such as *Simplicillium* and *Cladosporium* as potential antagonists of *H. vastatrix*. Wulansari et al. (2023) also isolated antagonistic fungi from coffee leaf rust-associated material in Indonesia and reported *Trichoderma atroviride* as a potential antagonist, possibly acting through mycoparasitism. These studies are useful for identifying local biocontrol resources, but they should be described as lesion-associated, phyllosphere-associated, or disease-microenvironment fungi unless endophytic colonization was confirmed.

A particularly promising example is *Cordyceps cateniannulata*. Pereira et al. (2024) isolated this fungus from coffee stem tissues and confirmed its endophytic colonization through reinoculation. The same fungus was also shown to parasitize the coffee leaf rust pathogen and infect coffee pests. This study is important because it demonstrates a multifunctional biocontrol profile involving endophytic colonization, pathogen parasitism, and insect pathogenicity. Such multifunctional strains may be valuable for integrated coffee pest and disease management, provided that their host compatibility, persistence, and non-target effects are carefully evaluated. Coffee endophytes and associated microorganisms involved in disease are listed in **Table 3**.

**Table 3:** Coffee endophytes and associated microorganisms involved in disease, nematode, and insect-pest suppression.

Functional category	Microbial taxon/ strain	Source/ tissue	Coffee species/ material	Proposed mechanism	Experimental evidence	Main outcome	Evidence strength	Reference
Coffee leaf rust suppression	Foliar fungal endophytes	Leaf endosphere	Two C. arabica varieties	Pre-emptive colonization and interference with rust establishment	Endophyte isolation and leaf-disc interaction assay	Some isolates reduced H. vastatrix colonization and disease severity	Direct coffee endophyte evidence; detached leaf assay	Angamarca et al., 2024
Antagonism against fungal and bacterial pathogens	Arthrinium, Biscogniauxia, Daldinia, Diaporthe, Nigrospora	Leaf endosphere	Coffee leaves	Antibiosis and/or pathogen inhibition	Endophyte isolation and in vitro antagonism assay	Several leaf endophytes showed strong antagonistic activity	Direct coffee endophyte screening; in vitro evidence	Li et al., 2022
Coffee leaf rust biocontrol	Endophytic bacteria	Coffee tissues	Coffee plants	Antagonism against H. vastatrix	Bioprospecting and disease-control screening	Identified bacterial endophytes with rust-control potential	Direct coffee endophytic bacterial evidence; direct control screening	Shiomi et al., 2006
Coffee tracheomycosis suppression	Endophytic Trichoderma spp.	Root endosphere	Healthy C. arabica roots	Antagonism, competition and possible mycoparasitism	Isolation and pathogen-control assay	Root endophytic Trichoderma spp. controlled coffee tracheomycosis	Direct coffee root-endophyte evidence; pathogen-control assay	Mulaw et al., 2013
Multifunctional biocontrol	Cordyceps cateniannulata	Stem endosphere	Coffee stem tissues	Endophytic colonization, rust parasitism, and insect pathogenicity	Isolation, reinoculation, and pathogen/pest assays	Confirmed endophyte that parasitized rust pathogen and infected coffee pests	Strong direct coffee endophyte evidence; multifunctional validation	Pereira et al., 2024
Nematode suppression	Bacillus pumilus, Bacillus mycoides	Root endosphere	Coffee roots	Antagonism against root-knot nematode; reduction of galling and reproduction	Isolation and pot experiment	Reduced gall formation and egg numbers of Meloidogyne incognita	Direct coffee root-endophyte evidence; pot-level validation	Mekete et al., 2009
Pest suppression / endophytic colonization	Beauveria bassiana	Introduced by stem injection, foliar spray, or soil drench; recovered from seedlings	Coffee seedlings	Endophytic colonization and possible insect-pathogenic protection	Inoculation and recovery assay	Colonized coffee seedlings, with higher recovery after stem injection; colonization declined over time	Induced endophytic colonization; recovery evidence; pest-control validation limited	Posada et al., 2007
Coffee leaf miner suppression and growth promotion	Metarhizium robertsii, M. brunneum	Coffee-cultivated soil; introduced by seed inoculation	Coffee plants/seedlings	Plant colonization, growth promotion, and insect pathogenicity	Seed inoculation and pest-damage assessment	Colonized coffee plants, promoted growth, and reduced coffee leaf miner damage	Plant-colonizing entomopathogenic fungus; greenhouse/cage application evidence	Martins et al., 2024

#### 4.2.2 Suppression of plant-parasitic nematodes and insect pests

Plant-parasitic nematodes, such as root-knot nematodes, can infect coffee roots, impair root development, reduce plant vigor, and cause yield losses, representing an important belowground biotic stress in coffee production (Saikai et al., 2023). Previous studies have shown that some endophytic bacteria derived from coffee plants have inhibitory potential against the southern root-knot nematode, *Meloidogyne incognita*, under pot conditions. Mekete et al. (2009) isolated 201 and 114 endophytic bacterial strains from coffee roots during the dry and rainy seasons, respectively, and found that *Bacillus pumilus* and *Bacillus mycoides* significantly reduced gall formation and egg numbers caused by *M. incognita*. These findings suggest that coffee root endophytic bacteria have potential value for the management of plant-parasitic nematodes. However, current studies in this field are still mainly limited to pot experiments or controlled conditions, and the stability of their field efficacy, as well as their long-term interactions with coffee roots, requires further validation.

In terms of insect pests, the coffee berry borer (*Hypothenemus hampei*) and coffee leaf miner (*Leucoptera coffeella*) are major pests affecting coffee yield and quality (Johnson et al., 2020; Dantas et al., 2021). Unlike the biological control of fungal diseases, microbial control strategies targeting insect pests must be interpreted through a multitrophic interaction model involving beneficial microorganisms, coffee plants, insect pests, and the surrounding environment (Pineda et al., 2013). Entomopathogenic fungi such as *Beauveria*, *Metarhizium*, and *Cordyceps* may suppress coffee pests through direct infection of insects, but their plant-colonizing or endophytic lifestyle may also create indirect plant-mediated effects.

After colonizing coffee tissues, entomopathogenic or endophytic fungi may alter plant defense signaling, cell-wall reinforcement, antioxidant systems, defensive enzyme activities, volatile organic compound production, and secondary metabolite profiles (Compant et al., 2010; Santoyo et al., 2016; Afzal et al., 2019). These changes may influence insect feeding, oviposition, larval development, or susceptibility to fungal infection. Such mechanisms are relevant to both the coffee berry borer and coffee leaf miner, but the strength of evidence differs among fungal taxa, inoculation methods, pest species, and experimental systems.

In coffee, particular attention has been given to whether entomopathogenic fungi can colonize coffee plants and extend their protective effects through an endophytic or plant-associated lifestyle. *Beauveria bassiana* was shown to colonize coffee seedlings after stem injection, foliar spraying, and soil drenching, with stem injection resulting in relatively higher recovery, although colonization declined over time (Posada et al., 2007). *Metarhizium robertsii* and *M. brunneum*, introduced through seed inoculation, colonized coffee plants, promoted plant growth, and reduced damage caused by the coffee leaf miner (Martins et al., 2024). Similarly, root drenching with *Metarhizium* sp. suspensions improved coffee seedling growth and reduced, to some extent, damage caused by *L. coffeella* (Franzin et al., 2022). A particularly strong multifunctional example is *Cordyceps cateniannulata*, which was isolated from coffee stem tissues, confirmed as an endophyte through reinoculation, and shown to parasitize the coffee leaf rust pathogen and infect coffee pests (Pereira et al., 2024).

Therefore, the evidence for endophyte-mediated pest suppression should be separated into three levels: (i) confirmed endophytic colonization combined with pest or pathogen suppression, as shown for multifunctional candidates such as *C. cateniannulata*; (ii) plant-colonizing entomopathogenic fungi introduced by seed inoculation, soil drenching, foliar application, or stem injection, such as *B. bassiana* and *Metarhizium* spp.; and (iii) external biocontrol agents applied against insect pests without demonstrated internal colonization. This distinction is important because external insect pathogenicity alone does not prove endophyte-mediated pest protection.

Compared with coffee leaf miner, direct evidence for endophyte-mediated suppression of the coffee berry borer remains more limited because *H. hampei* develops and feeds inside coffee berries. Therefore, future studies should test whether fruit-, seed-, or stem-colonizing endophytes can alter berry chemistry, volatile cues, insect entry behavior, larval development, or susceptibility to entomopathogenic fungi.

**Figure 4:**
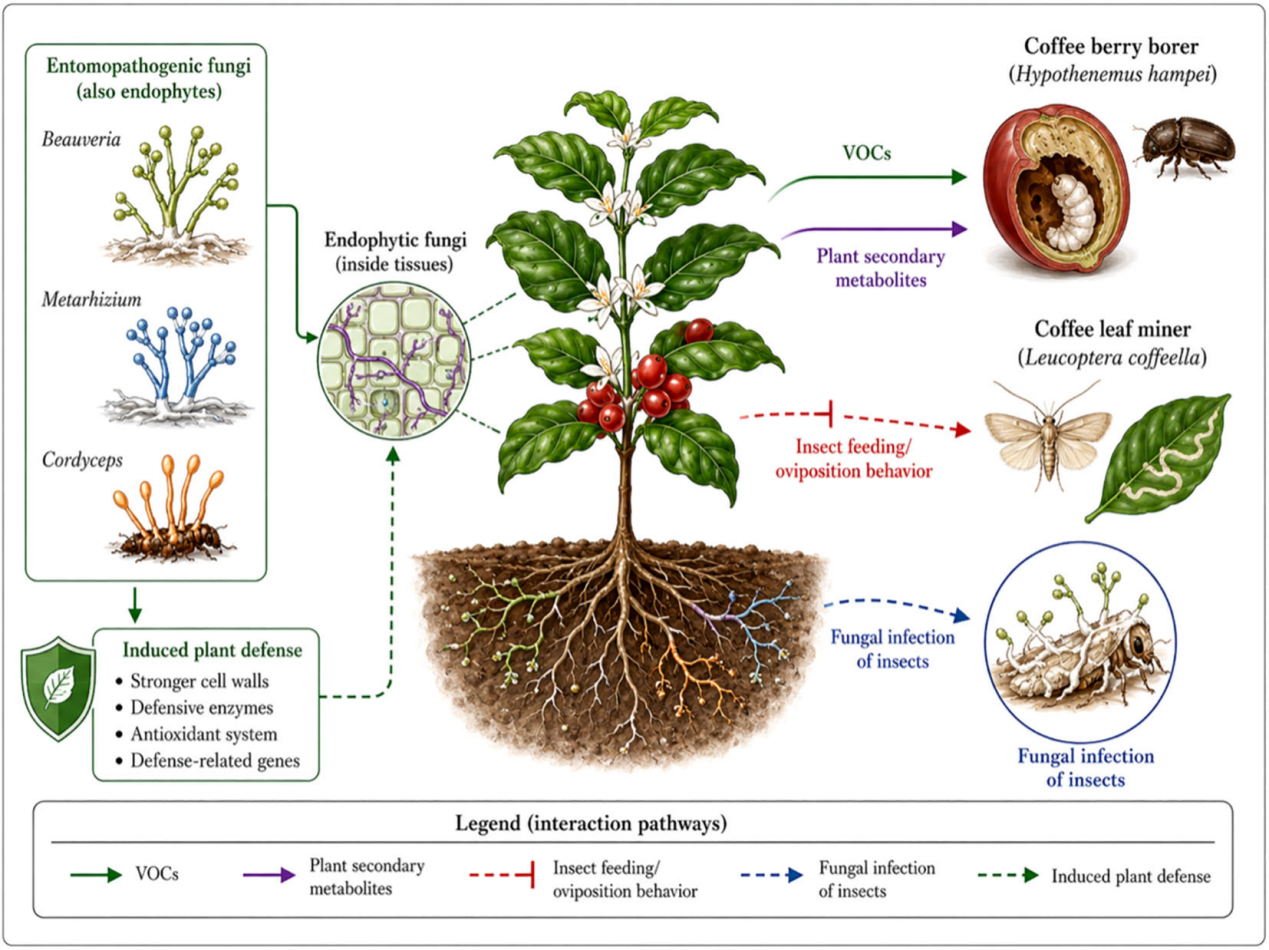
Proposed multitrophic interactions among coffee plants, endophytic or entomopathogenic fungi, insect pests, and induced plant defense responses. *Beauveria*, *Metarhizium*, and *Cordyceps* may colonize coffee tissues and contribute to pest suppression through both direct fungal infection and plant-mediated responses. Fungal colonization may induce cell-wall strengthening, defensive enzymes, antioxidant systems, defense-related genes, volatile organic compounds, and secondary metabolites, which may influence insect feeding and oviposition behavior. These interactions are especially relevant to *H. hampei* and *L. coffeella*, but further experimental and field validation is needed.

In addition to studies focusing on plant colonization, entomopathogenic fungi such as *B. bassiana* and *Metarhizium* spp. also show application potential in the biological control and integrated management of the coffee berry borer (Hollingsworth et al., 2020; Bayman et al., 2021; Wraight et al., 2022; Chang et al., 2023; Cruz et al., 2024). Overall, beneficial microorganisms, including coffee endophytic bacteria, *Beauveria*, and *Metarhizium*, have promising value for the control of plant-parasitic nematodes and insect pests in coffee. Nevertheless, their efficacy may be affected by strain origin, inoculation method, coffee genotype, pest development stage, colonization stability, field environment, and compatibility with existing pest-management practices. Therefore, long-term field validation, colonization tracking, non-target assessment, and optimization of application strategies are still needed. A proposed model of multitrophic interactions among coffee plants, endophytic or entomopathogenic fungi, insect pests, and induced plant defense responses is shown in **Figure 4**. Coffee endophytes and associated microorganisms involved in nematode and insect-pest suppression are listed in **Table 3**.

### 4.3 Fruit- and seed-associated endophytes in coffee quality formation

Coffee fruits and seeds harbor tissue-specific bacterial and fungal communities (Mahatmanto et al., 2023; Oliveira et al., 2013; Tenea et al., 2025; Vaughan et al., 2015; Vega et al., 2008). These microorganisms are not only closely associated with the microenvironments of fruits and seeds, but may also serve as part of the initial microbial sources during coffee bean fermentation (Vaughan et al., 2015; Mahatmanto et al., 2023). During post-harvest fermentation, microbial community succession can influence sugar metabolism, organic acid formation, and changes in volatile metabolites (De Bruyn et al., 2016; Haile and Kang, 2019; Kutos et al., 2025; Shen et al., 2024). Studies on natural, self-induced anaerobic, and anaerobic fermentation have also shown that differences in microbial communities, altitude, and processing methods are correlated with coffee chemical composition and sensory quality (Cruz-O’Byrne et al., 2021; Martinez et al., 2022; Pereira et al., 2022; Pothakos et al., 2020; Todhanakasem et al., 2024). Overall, the composition of fruit- and seed-associated microbiota varies with host species, cultivation environment, maturity stage, and post-harvest processing method, and these variations may ultimately influence flavor perception (Kutos et al., 2025; Tenea et al., 2025).

Mahatmanto et al. (2023) analyzed the endophytic bacterial communities in the fruits of *C. arabica* L. and *C. canephora* Pierre ex A. Froehner and found that both coffee species harbored fermentation-associated bacterial groups, including lactic acid bacteria, acetic acid bacteria, and Enterobacteria. In *C. arabica* fruits, *Leuconostoc pseudomesenteroides* was dominant, suggesting that the indigenous microbiota present at the fresh fruit stage may influence the direction of subsequent fermentation. Tenea et al. (2025) further showed that microbial diversity and functional characteristics differed markedly among fruits of different maturity stages and *C. arabica* varieties, indicating that fruit ripening itself can reshape the microbial basis associated with coffee quality and safety.

During post-harvest natural fermentation, fruit- and seed-associated microorganisms may act together with microorganisms introduced from the processing environment to participate in mucilage degradation, sugar metabolism, and the transformation of aroma-related compounds, thereby affecting fermentation efficiency and flavor development (Sakiyama et al., 2001; De Bruyn et al., 2016; Haile and Kang, 2019; Kutos et al., 2025). Cruz-O’Byrne et al. (2021) used high-throughput sequencing to characterize microbial communities during the natural fermentation of *C. arabica* in northern Colombia. They found that fungal richness was higher than bacterial richness, whereas fungal community diversity was lower than that of bacteria. The bacterial community was mainly composed of lactic acid bacteria, *Leuconostoc*, and acetic acid bacteria, while the fungal community was dominated by *Kazachstania* and two unclassified fungal taxa. These results suggest that microbial diversity during fermentation may ultimately affect coffee cup quality. Pothakos et al. (2020) conducted a time-series metagenomic analysis of wet fermentation of *C. arabica* in Ecuador and showed that the process consisted of successive microbial succession stages, with lactic acid bacteria becoming dominant in the middle and late stages, accompanied by functional shifts related to sugar metabolism and cell wall degradation. Martínez et al. (2022), through molecular, chemical, and sensory analyses, found that differences in microbial communities under different fermentation conditions were associated with variations in chemical composition and cup quality.

From a more specific perspective of flavor formation, Todhanakasem et al. (2024) investigated wet fermentation of coffee in Thailand and found that dominant microbial communities during fermentation were significantly correlated with several desirable flavor and aroma compounds, some of which may also have health-related relevance. Meanwhile, Pereira et al. (2022) showed that self-induced anaerobic fermentation could simultaneously affect microbial communities, chemical composition, and sensory quality.

At present, most studies on coffee quality formation remain focused on the analysis of fruit-associated microbial communities, the tracking of natural fermentation processes, and their correlations with quality-related phenotypes. In contrast, studies involving isolation and re-inoculation of fruit- or seed-derived endophytic microorganisms, colonization tracking, and direct quality validation remain relatively limited. Overall, endophytic microorganisms in coffee fruits and seeds may contribute to quality formation by influencing the composition of the initial microbiota in natural fermentation and the subsequent metabolic processes. However, their specific mechanisms of action still require further validation.

## 5 Bioprospecting and application-oriented development of coffee endophytes

Coffee endophyte resource development refers to the isolation, characterization, preservation, and application-oriented evaluation of beneficial microorganisms associated with coffee tissues. These microbial resources can support cultivation-stage applications, including nutrient mobilization, plant growth promotion, stress tolerance, disease suppression, and pest management (Duong et al., 2021; Mekete et al., 2009; Pereira et al., 2024; Shiomi et al., 2006; Silva et al., 2012). They may also contribute to postharvest and quality-oriented applications through fruit- and seed-associated microbiomes, pectin degradation, fermentation-related metabolite transformation, caffeine degradation, and flavor modulation (De Bruyn et al., 2016; Kutos et al., 2025; Mahatmanto et al., 2023; Sakiyama et al., 2001; Tenea et al., 2025). Therefore, the value of coffee endophytes should be assessed not only by their occurrence in coffee tissues, but also by their colonization ability, functional stability, biosafety, and suitability for specific production or processing systems (Compant et al., 2010; Backer et al., 2018; Duong et al., 2020; Gómez et al., 2021). Representative studies supporting these utilization pathways are summarized in **Table 4**.

**Table 4:** Representative studies on the resource development and utilization potential of coffee endophytes.

Study focus	Microbial resource	Utilization pathway	Main outcome	Application relevance	Validation level and gap	Reference
Discovery of culturable bacterial endophytes	Endophytic bacteria from C. arabica	Strain-resource discovery and functional exploration	Reported culturable bacterial endophytes from coffee tissues	Provides early basis for coffee bacterial endophyte collections	Discovery-level evidence; functional and application validation limited	Vega et al., 2005
Growth promotion and coffee leaf rust biocontrol	Endophytic microorganisms from coffee tissues	Biofertilizer and biocontrol screening	Endophytes showed plant growth-promoting and coffee leaf rust biocontrol potential	Strong selection of cultivation-stage microbial resources	Controlled-condition evidence; broader field and formulation validation needed	Silva et al., 2012
Multifunctional bacterial endophytes	Vietnamese coffee bacterial endophytes	Antifungal and nematicidal biocontrol screening	Endophytic bacteria displayed in vitro antifungal and nematicidal activities	Useful for selecting multifunctional strains for disease/nematode management	Mostly in vitro evidence; plant-performance, field efficacy, and colonization stability need validation	Duong et al., 2021
Coffee leaf rust biocontrol	Endophytic bacteria from coffee tissues	Biological control of Hemileia vastatrix	Identified bacterial endophytes with potential to suppress coffee leaf rust	Directly relevant to rust-management bioproduct development	Screening-level evidence; greenhouse/field confirmation and formulation testing needed	Shiomi et al., 2006
Coffee tracheomycosis control	Endophytic Trichoderma spp. from healthy C. arabica roots	Root-endophyte-based disease control	Root endophytic Trichoderma spp. controlled coffee tracheomycosis	Supports use of root endophytes for vascular/root disease management	Controlled pathogen-assay evidence; field testing and strain-specific safety assessment needed	Mulaw et al., 2013
Endophytic entomopathogen establishment	Beauveria bassiana	Inoculation of coffee plants for endophytic colonization	B. bassiana colonized coffee seedlings after stem injection, foliar spray, and soil drench	Supports use of entomopathogenic fungi as plant-colonizing pest-control agents	Colonization-recovery evidence; colonization declined over time and pest-control efficacy requires stronger validation	Posada et al., 2007
Nematode suppression	Bacillus pumilus and Bacillus mycoides from coffee roots	Biological control of Meloidogyne incognita	Reduced gall formation and egg numbers under pot conditions	Useful for developing bionematicide/endophyte-based root protection	Pot-level evidence; field persistence and efficacy remain uncertain	Mekete et al., 2009
Multifunctional biocontrol	Cordyceps cateniannulata from coffee stems	Integrated disease and pest management	Confirmed endophytic colonization; parasitized coffee leaf rust and infected coffee pests	Strong candidate for multifunctional biocontrol development	Strong experimental evidence; host safety, non-target effects, field persistence, and performance require validation	Pereira et al., 2024
Coffee seed inoculation for growth promotion and coffee leaf miner biocontrol	Metarhizium robertsii RD-20.114 and M. brunneum RD-20.120	Seed inoculation of C. arabica with fungal conidia	Inoculated seedlings showed fungal recovery, delayed Leucoptera coffeella development, reduced egg laying in the next generation, and improved growth trait	Demonstrates a practical nursery-stage inoculation route compatible with seedling production	Greenhouse/cage evidence; field persistence and mechanism require validation	Martins et al., 2024
Root-associated pest suppression	Metarhizium sp. associated with coffee seedling roots	Root drenching for growth promotion and leaf miner protection	Improved seedling growth and reduced Leucoptera coffeella damage	Supports root-zone application of entomopathogenic fungi	Greenhouse/controlled evidence; endophytic status and long-term persistence should be clarified	Franzin et al., 2022
Postharvest enzyme resource	Endophytic strain isolated from coffee cherries	Pectinase/pectin lyase production for processing-related use	Characterized pectin lyase from a coffee-cherry endophytic strain	Relevant to mucilage degradation and postharvest bioprocessing	Enzyme-level evidence; not directly validated in coffee fermentation system	Sakiyama et al., 2001
Fruit microbiome and quality potential	Indigenous bacteria from Arabica and Robusta coffee cherries	Quality-oriented microbial resource exploration	Identified indigenous bacteria with presumptive impact on coffee quality	Supports fruit-associated microbial screening for quality applications	Correlative fruit-microbiome evidence; endophytic status, reinoculation, and sensory validation needed	Mahatmanto et al., 2023
Bioactive-metabolite bioprospecting	Colletotrichum siamense 165b, an endophytic fungus isolated from C. arabica cv. IAPAR-59	Secondary-metabolite discovery for antibacterial and antitumor potential	Crude fungal extract showed antibacterial and cytotoxic activities, and several metabolites were detected	Expands the resource value of coffee endophytes beyond cultivation-oriented use	Bioactivity-screening evidence; not validated for coffee growth, disease control, stress tolerance, pest suppression, or quality improvement	do Espírito Santo et al., 2023

### 5.1 Isolation, screening, and preservation of functional strains

The development of coffee endophyte resources begins with the reliable recovery of culturable strains from different plant tissues, including roots, stems, leaves, fruits, and seeds. Standard procedures generally include tissue collection, surface sterilization, tissue sectioning or grinding, culture-based isolation, purification, molecular identification, functional screening, and where possible, host reinoculation or colonization validation (Compant et al., 2010; Duong et al., 2021; Li et al., 2022; Santoyo et al., 2016). The representativeness of recovered strains is strongly influenced by the intensity of surface sterilization, tissue type, sampling season, coffee genotype, plant health status, and culture medium (Silva et al., 2012; Duong et al., 2021; Zhang et al., 2025). Therefore, isolation protocols should be designed according to the intended application target rather than applied as a uniform procedure for all tissues.

Tissue origin is important for resource screening. Root- and stem-derived endophytes may be more relevant for nutrient acquisition, drought tolerance, vascular colonization, and root protection, whereas leaf endophytes are more closely linked with foliar disease suppression and phyllosphere interaction (Angamarca et al., 2024; Mulaw et al., 2013; Pratiwi et al., 2020; Shiomi et al., 2006). Fruit- and seed- associated endophytes may be more suitable for studies related to seed microbiomes, postharvest fermentation, pectin degradation, and quality-associated metabolite transformation (Oliveira et al., 2013; Tenea et al., 2025; Vaughan et al., 2015). This tissue-specific distinction can improve the selection of candidate strains for different utilization pathways.

Functional screening should move beyond single-trait evaluation. Although phosphate-solubilization halos, IAA production, siderophore formation, ACC deaminase activity, enzyme assays, and inhibition-zone assays are useful preliminary indicators, they do not necessarily predict performance inside coffee plants or under field conditions (Silva et al., 2012; Pratiwi et al., 2020; Duong et al., 2021). For a perennial woody crop such as coffee, candidate strains should preferably be screened for multiple complementary traits, such as nutrient mobilization, phytohormone regulation, stress mitigation, pathogen antagonism, nematode or insect suppression, and host-colonization ability (Duong et al., 2021; Franzin et al., 2022; Martins et al., 2024; Pereira et al., 2024). This approach is more useful than selecting strains only on the basis of one strong *in vitro* response.

Preservation of elite strains is also an important part of resource development. Promising isolates should be maintained in well-documented microbial collections with information on tissue origin, coffee species or cultivar, geographic location, isolation method, molecular identity, functional traits, and reinoculation results. Such strain banks can support future comparative studies, synthetic community construction, formulation development, and region-specific microbial inoculant design (Compant et al., 2010; Backer et al., 2018; Lopez et al., 2025; Zhang et al., 2025).

### 5.2 Cultivation-stage utilization of coffee endophytes

At the cultivation stage, coffee endophytes can be developed as biological resources to improve seedling establishment, nutrient-use efficiency, stress tolerance, and plant protection. Growth-promoting coffee endophytes and closely associated plant growth-promoting microorganisms may contribute through nitrogen fixation, phosphorus solubilization, siderophore-mediated iron acquisition, phytohormone production, and improved root development (Jiménez-Salgado et al., 1997; Silva et al., 2012; Muleta et al., 2013; Teshome et al., 2017; Kunwar et al., 2018; Pratiwi et al., 2020; Duong et al., 2021; Jasso-Arreola et al., 2025). These functions are particularly relevant during nursery production, where early root growth and seedling vigor strongly influence later field performance.

Biocontrol is another important utilization pathway. Coffee-associated bacterial and fungal endophytes have been evaluated for suppression of coffee leaf rust, coffee tracheomycosis, and other fungal pathogens (Li et al., 2022; Mulaw et al., 2013; Pereira et al., 2024; Shiomi et al., 2006). Endophytic bacteria from coffee roots have also shown potential against *Meloidogyne incognita*, while plant-colonizing entomopathogenic fungi such as *Beauveria bassiana*, *Cordyceps cateniannulata*, and *Metarhizium* spp. have been studied for pest suppression and plant-growth benefits (Martins et al., 2024; Mekete et al., 2009; Pereira et al., 2024; Posada et al., 2007). These examples indicate that coffee endophyte utilization should not be limited to biofertilizer development, but can also contribute to integrated disease and pest management.

Practical application can be designed through nursery substrate inoculation, seed or seedling treatment, root dipping, soil drenching, foliar application, or integration with organic amendments. Nursery-stage inoculation may be particularly suitable for coffee because seedlings can be treated under controlled conditions, colonization can be monitored before transplanting, and candidate strains can be selected before exposure to field-level stress (Posada et al., 2007; Martins et al., 2024). However, application strategies should be adapted to coffee variety, soil type, altitude, shade management, fertilization practice, pest pressure, and local production system (Bilen et al., 2022; Duong et al., 2020; Gómez et al., 2021; Zhao et al., 2024). Therefore, locally adapted strains or carefully selected microbial consortia may be more effective than broadly applied single-strain inoculants.

### 5.3 Postharvest and quality-oriented utilization

Fruit- and seed-associated endophytes may also contribute to postharvest processing and quality-oriented applications. Coffee cherries and seeds contain microbial communities that can interact with fermentation microbiota and influence mucilage degradation, metabolite conversion, aroma precursor formation, and flavor-related pathways (Mahatmanto et al., 2023; Oliveira et al., 2013; Tenea et al., 2025; Vaughan et al., 2015). Processing-related microbial functions, including pectin degradation and metabolite transformation, have also been linked with coffee fermentation and quality development (Sakiyama et al., 2001; De Bruyn et al., 2016; Kutos et al., 2025). Pectin-degrading endophytic strains isolated from coffee cherries also indicate that internal fruit-associated microorganisms may provide enzymes relevant to processing-related biotransformation (Sakiyama et al., 2001). However, this area should be interpreted carefully because many quality-related studies focus on fermentation microbiota or fruit-surface communities rather than strictly confirmed endophytes (De Bruyn et al., 2016; Kutos et al., 2025; Shen et al., 2024).

For quality-oriented utilization, the key question is whether fruit- or seed-associated endophytes can produce reproducible effects under controlled fermentation or postharvest processing. Candidate strains should therefore be evaluated using controlled fermentation trials, metabolomic profiling, volatile-compound analysis, sensory evaluation, and comparison across coffee varieties and processing methods (Kutos et al., 2025; Pereira et al., 2022; Todhanakasem et al., 2024). This is necessary to distinguish direct endophyte effects from broader effects of spontaneous fermentation communities, processing environment, starter cultures, and cherry maturity.

Endophyte-based quality improvement may be particularly relevant for specialty coffee production, where local microbial communities can contribute to flavor differentiation and terroir-associated quality traits (Kutos et al., 2025; Mahatmanto et al., 2023; Wang et al., 2024). Microbial caffeine degradation and flavor modulation have also attracted attention, but such evidence should not be interpreted as direct endophyte-mediated quality improvement unless the microbial strains are traced to fruit or seed endophytic communities (Ran et al., 2025). Therefore, postharvest utilization of coffee endophytes should focus on strain origin, safety, metabolic function, fermentation compatibility, and reproducible sensory outcomes.

### 5.4 Validation and product development pipeline

For coffee endophytes to move from discovery to practical application, a step-wise validation pipeline is required. This pipeline should include strain identification, functional screening, host reinoculation, colonization confirmation, biosafety assessment, greenhouse evaluation, field testing, formulation development, and application-method optimization (Backer et al., 2018; Compant et al., 2010; Duong et al., 2020; Gómez et al., 2021). Colonization confirmation is particularly important because an isolate cannot be considered a functional endophyte-based resource unless it can enter, persist, or become re-established within coffee tissues after application (Martins et al., 2024; Pereira et al., 2024; Posada et al., 2007).

The long growth cycle and complex ecology of coffee make validation more difficult than in many annual crops. Coffee plants remain in the field for many years and are exposed to seasonal changes, variable shade conditions, soil heterogeneity, pest and disease pressure, and different management histories (Bilen et al., 2022; Duong et al., 2020; Worku, 2023; Zhao et al., 2024). Candidate strains should therefore be tested not only for short-term effects, but also for persistence, functional stability, host compatibility, and interaction with resident microbiomes (Compant et al., 2010; Duong et al., 2020; Gómez et al., 2021). Multi-season and multi-location validation will be particularly important for strains intended for commercial or farmer-level use.

Formulation is another key requirement for utilization. Effective microbial products require suitable carriers, shelf life, stress tolerance during storage, and practical application methods (Backer et al., 2018; Duong et al., 2020; Gómez et al., 2021). For cultivation-stage use, coffee inoculants could be developed for nursery substrates, seedling-root dipping, soil drenching, organic fertilizer enrichment, or integrated pest and disease management. For quality-oriented use, candidate strains should be developed as processing or fermentation agents only after confirming their safety, metabolic activity, fermentation compatibility, and reproducible effects on sensory quality (De Bruyn et al., 2016; Kutos et al., 2025; Ran et al., 2025; Sakiyama et al., 2001; Tenea et al., 2025).

Overall, the development of coffee endophyte resources should connect tissue-specific isolation, multifunctional screening, host-colonization validation, multi-environment testing, and product-oriented formulation. This pathway can help shift coffee endophyte research from descriptive community surveys toward practical microbial tools for sustainable cultivation and quality-oriented processing.

## 6 Limitations, biosafety, and future research priorities

Although coffee endophytes show considerable potential for growth promotion, nutrient mobilization, biological control, stress tolerance, and quality-related applications, several limitations still restrict their practical use. The main challenges include weak field validation, limited mechanistic evidence, uncertain colonization stability, strain-specific functional variation, biosafety concerns, and insufficient product-development studies. These limitations should be addressed before coffee endophytes can be reliably used in sustainable cultivation or postharvest processing.

### 6.1 Evidence gaps and field-validation bottlenecks

Despite increasing interest in coffee endophytes, most available studies remain concentrated on strain isolation, *in vitro* functional screening, pot experiments, or short-term greenhouse validation. Field-level evidence remains limited, particularly for long-term colonization, persistence, and functional stability under commercial coffee-production conditions (Duong et al., 2020; Gómez et al., 2021; Asad et al., 2023; Lopez et al., 2025). This creates a major bottleneck between promising laboratory results and practical field application.

Many studies still rely on single-site sampling, single-time-point surveys, or short-term validation. This limits our understanding of how endophytic communities fluctuate across seasons, plant developmental stages, coffee genotypes, altitudes, and management systems (Duong et al., 2020; Gómez et al., 2021; Asad et al., 2023). Long-term and multi-location studies are needed to identify stable beneficial taxa and distinguish core endophytes from transient microorganisms.

A major limitation in the field is the gap between laboratory screening and field application. Traits such as phosphate solubilization, IAA production, siderophore production, or pathogen inhibition are often measured under controlled conditions, but these traits may not be expressed consistently inside coffee plants or under variable field environments. Therefore, laboratory traits should be treated as preliminary indicators rather than direct predictors of field performance.

Coffee poses additional challenges because it is a perennial woody crop with a long production cycle, diverse tissue niches, and strong environmental variation among production systems. Candidate strains must therefore be evaluated across seasons, varieties, soil types, altitudes, shade conditions, and management practices before they can be recommended for practical use.

### 6.2 Biosafety, host specificity, and regulatory concerns

Biosafety assessment is essential before coffee endophytes are developed as bioinoculants or postharvest microbial resources. Some genera reported as coffee-associated endophytes may contain beneficial strains as well as opportunistic pathogens, toxin producers, or plant pathogens. Therefore, application should not be based on genus-level identity alone. Candidate strains should be evaluated at the strain level using pathogenicity tests, genome-based safety screening, antibiotic-resistance profiling, and assessment of undesirable metabolite production.

Host specificity is another important concern. A strain isolated from one coffee genotype, tissue, region, or management system may not perform equally well in another production context. Candidate endophytes should therefore be tested for compatibility with different coffee genotypes, tissue targets, environmental conditions, and resident microbial communities. This is especially important for strains intended for broad commercial use, because poor host compatibility may reduce colonization, persistence, or functional expression.

Regulatory and ecological risks should also be considered. Introduced microbial inoculants may interact with native microbiota, non-target organisms, and existing pest- or disease-management practices. For postharvest use, safety concerns are even more direct because microbial strains may influence fermentation products and final beverage quality. Thus, future application-oriented studies should include strain traceability, non-target assessment, toxin and metabolite screening, and compliance with local biofertilizer, biopesticide, or food-processing regulations.

### 6.3 Future research pathway

Although sequencing, metagenomics, and multi-omics approaches have improved our understanding of coffee-associated microbial communities, the causal links between microbiome structural shifts and plant growth, stress resistance, disease suppression, or quality formation remain poorly resolved (De Bruyn et al., 2016; Trivedi et al., 2020; Xiong et al., 2021; Shen et al., 2024; Kutos et al., 2025; Wu et al., 2025). Future studies should combine microbiome profiling with host phenotyping, metabolomics, transcriptomics, and controlled inoculation experiments to identify functional mechanisms rather than only community patterns.

Future research should integrate core microbiome identification, elite strain screening, synthetic community construction, colonization tracking, multi-environment validation, and formulation development. A useful research pathway should include: (i) tissue-specific isolation and sequencing; (ii) evidence-level classification of candidate microorganisms; (iii) functional screening under controlled conditions; (iv) reinoculation and colonization tracking in coffee plants; (v) physiological, molecular, and metabolomic validation of mechanisms; (vi) greenhouse and field testing across environments; and (vii) formulation, biosafety, and application-method optimization. This integrated pathway would help move coffee endophyte research from descriptive surveys toward reproducible, application-oriented microbial technologies.

## 7 Conclusions and perspectives

Coffee endophytes represent an important component of the coffee plant microbiome, with high diversity, tissue specificity, and functional potential across roots, stems, leaves, fruits, and seeds. Current evidence indicates that endophytic bacteria and fungi can support coffee growth, stress adaptation, and plant health through nutrient mobilization, phytohormone regulation, induced resistance, pathogen antagonism, and modulation of host metabolism. Fruit- and seed-associated microbial communities may also contribute to postharvest microbial processes linked with fermentation and quality formation, although these effects should be distinguished from confirmed endophyte-mediated functions.

Overall, coffee endophytes provide a valuable entry point for understanding coffee–microbiome interactions and offer promising microbial resources for sustainable cultivation, low-input disease and pest management, and quality-oriented processing. However, their practical use depends on stronger evidence connecting microbial identity, host colonization, functional expression, and stable performance under real production conditions. Therefore, future research should focus on identifying core beneficial taxa, validating multifunctional strains, tracking colonization within coffee tissues, and evaluating microbial inoculants or consortia across seasons, sites, coffee genotypes, and management systems. By integrating culture-based isolation, microbiome profiling, host phenotyping, multi-omics analysis, biosafety assessment, formulation development, and field-level validation, coffee endophyte research can move from descriptive diversity studies toward reliable application-oriented strategies. Such advances will help develop microbial tools that support sustainable coffee production, ecological crop protection, and improved quality in coffee processing.

## References

[B1] Afzal I. Shinwari Z. K. Sikandar S. Shahzad S. (2019). Plant beneficial endophytic bacteria: mechanisms, diversity, host range and genetic determinants. Microbiol. Res. 221, 36–49. doi: 10.1016/j.micres.2019.02.001 30825940

[B2] Angamarca R. A. P. Rojas J. R. Rodríguez A. S. González M. X. R. (2024). Diversity of leaf fungal endophytes from two Coffea arabica varieties and antagonism towards coffee leaf rust. Plants 13, 814. doi: 10.3390/plants13060814 38592839 PMC11154406

[B500] Asad S. Priyashantha A. K. H. Tibpromma S. Luo Y. Zhang J. Fan Z. . (2023). Coffee-associated endophytes: plant growth promotion and crop protection. Biology 12, 911. doi: 10.3390/biology12070911 37508343 PMC10376224

[B501] Backer R. Rokem J. S. Ilangumaran G. Lamont J. Praslickova D. Ricci E. . (2018). Plant growth-promoting rhizobacteria: context, mechanisms of action, and roadmap to commercialization of biostimulants for sustainable agriculture. Front. Plant Sci. 9, 1473. doi: 10.3389/fpls.2018.01473 30405652 PMC6206271

[B3] Bayman P. Marino Y. A. Rodríguez N. M. G. Sierra O. F. O. Rehner S. A. (2021). Local isolates of Beauveria bassiana for control of the coffee berry borer Hypothenemus hampei in Puerto Rico: virulence, efficacy and persistence. Biol. Control 155, 104533. doi: 10.1016/j.biocontrol.2021.104533 38826717

[B4] Bilen C. De Chami C. Mereu V. Trabucco A. Marras S. Spano D. (2022). A systematic review on the impacts of climate change on coffee agrosystems. Plants 12, 102. doi: 10.3390/plants12010102 36616231 PMC9824350

[B5] Bunn C. Läderach P. Rivera O. O. Kirschke D. (2015). A bitter cup: climate change profile of global production of Arabica and Robusta coffee. Clim. Change 129, 89–101. doi: 10.1007/s10584-014-1306-x 30311153

[B6] Castillo N. E. T. Martinez E. M. M. Sierra J. S. O. Mendoza R. A. R. Saldivar R. P. Iqbal H. M. N. (2020). Impact of climate change and early development of coffee rust: an overview of control strategies to preserve organic cultivars in Mexico. Sci. Total Environ. 738, 140225. doi: 10.1016/j.scitotenv.2020.140225 32806380

[B7] Chang F. M. Lu H. L. Nai Y. S. (2023). Evaluation of potential entomopathogenic fungus, Beauveria bassiana, for controlling the coffee berry borer Hypothenemus hampei (Ferrari) (Coleoptera: Curculionidae) in Taiwan. J. Asia-Pac. Entomol. 26, 102118. doi: 10.1016/j.aspen.2023.102118 38826717

[B8] Compant S. Clément C. Sessitsch A. (2010). Plant growth-promoting bacteria in the rhizo- and endosphere of plants: their role, colonization, mechanisms involved and prospects for utilization. Soil Biol. Biochem. 42, 669–678. doi: 10.1016/j.soilbio.2009.11.024 38826717

[B9] Cruz M. O. Bardales J. M. S. Espinoza S. T. L. Cruz C. O. Fasabi L. D. M. Contreras L. J. (2024). Compatibility of native strains of Beauveria Peruviensis and Metarhizium sp. as strategy for biological control of coffee berry borer (Hypothenemus hampei, Ferrari). Agronomy 14, 904. doi: 10.3390/agronomy14050904 30654563

[B10] Cruz-O’Byrne R. Piraneque-Gambasica N. Aguirre-Forero S. (2021). Microbial diversity associated with spontaneous coffee bean fermentation process and specialty coffee production in northern Colombia. Int. J. Food Microbiol. 354, 109282. doi: 10.1016/j.ijfoodmicro.2021.109282 34140187

[B11] DaMatta F. M. Martins S. C. V. Ramalho J. D. C. (2025). “ Ecophysiology of coffee growth and production in a context of climate changes,” in Coffee - A Glimpse into the Future. Advances in Botanical Research, vol. 114. (United States: Academic Press), 97–139. doi: 10.1016/bs.abr.2024.07.004

[B12] Dantas J. Motta I. O. Vidal L. A. Nascimento E. F. M. B. Bílio J. Pupe J. M. . (2021). A comprehensive review of the coffee leaf miner Leucoptera coffeella (Lepidoptera: Lyonetiidae): a major pest for the coffee crop in Brazil and other Neotropical countries. Insects 12, 1130. doi: 10.20944/preprints202010.0629.v1 34940218 PMC8707027

[B13] Davis A. P. Gole T. W. Baena S. Moat J. (2012). The impact of climate change on indigenous Arabica coffee (Coffea arabica): predicting future trends and identifying priorities. PloS One 7, e47981. doi: 10.1371/journal.pone.0047981 23144840 PMC3492365

[B14] Davis A. P. Govaerts R. Bridson D. M. Stoffelen P. (2006). An annotated taxonomic conspectus of the genus Coffea (Rubiaceae). Bot. J. Linn. Soc 152, 465–512. doi: 10.1111/j.1095-8339.2006.00584.x 40046247

[B15] De Bruyn F. Zhang S. J. Pothakos V. Torres J. Lambot C. Moroni A. V. . (2016). Exploring the impact of post-harvest processing on the microbiota and metabolite profiles during a case of green coffee bean production. Appl. Environ. Microbiol. 83, e02398-16. doi: 10.1128/aem.02398-16 27793826 PMC5165123

[B16] do Espírito Santo B. C. Oliveira J. A. D. S. Ribeiro M. A. D. S. Schoffen R. P. Polli A. D. Polonio J. C. . (2023). Antitumor and antibacterial activity of metabolites of endophytic Colletotrichum siamense isolated from coffee (Coffea arabica L. cv. IAPAR-59). Braz. J. Microbiol. 54, 2651–2661. doi: 10.1007/s42770-023-01104-0 37642890 PMC10689633

[B17] Duong B. Marraccini P. Maeght J. L. Vaast P. Lebrun M. Duponnois R. (2020). Coffee microbiota and its potential use in sustainable crop management: a review. Front. Sustain. Food. Syst. 4, 607935. doi: 10.3389/fsufs.2020.607935

[B18] Duong B. Nguyen H. X. Phan H. V. Colella S. Trinh P. Q. Hoang G. T. . (2021). Identification and characterization of Vietnamese coffee bacterial endophytes displaying *in vitro* antifungal and nematicidal activities. Microbiol. Res. 242, 126613. doi: 10.1016/j.micres.2020.126613 33070050

[B19] Franzin M. L. Moreira C. C. Silva L. N. P. D. Martins E. F. Fadini M. A. M. Pallini A. . (2022). Metarhizium associated with coffee seedling roots: positive effects on plant growth and protection against Leucoptera coffeella. Agriculture 12, 2030. doi: 10.3390/agriculture12122030

[B20] Fulthorpe R. Martin A. R. Isaac M. E. (2020). Root endophytes of coffee (Coffea arabica): variation across climatic gradients and relationships with functional traits. Phytobiomes J. 4, 27–39. doi: 10.1094/pbiomes-04-19-0021-r

[B21] Ge Y. Zhang F. Y. Xie C. Qu P. Jiang K. L. Du H. B. . (2023). Effects of different altitudes on Coffea arabica rhizospheric soil chemical properties and soil microbiota. Agronomy 13, 471. doi: 10.3390/agronomy13020471 30654563

[B22] Glick B. R. (2014). Bacteria with ACC deaminase can promote plant growth and help to feed the world. Microbiol. Res. 169, 30–39. doi: 10.1016/j.micres.2013.09.009 24095256

[B23] Gómez N. U. Salem M. E. A. Lojan P. Encalada M. Hurtado L. Araujo S. . (2021). Plant growth-promoting microorganisms in coffee production: from isolation to field application. Agronomy 11, 1531. doi: 10.3390/agronomy11081531

[B24] González H. C. Bloomberg J. Picado E. A. Yarwood S. Chaverri P. (2024). Agricultural practices influence foliar endophytic communities in coffee plants of different varieties. Agrosyst. Geosci. Environ. 7, e20476. doi: 10.1002/agg2.20476

[B25] Haile M. Kang W. H. (2019). The role of microbes in coffee fermentation and their impact on coffee quality. J. Food Qual. 2019, 4836709. doi: 10.1155/2019/4836709

[B26] Hollingsworth R. G. Aristizábal L. F. Shriner S. Mascarin G. M. Moral R. A. Arthurs S. P. (2020). Incorporating Beauveria bassiana into an integrated pest management plan for coffee berry borer in Hawaii. Front. Sustain. Food. Syst. 4, 22. doi: 10.3389/fsufs.2020.00022

[B27] Jaramillo J. Muchugu E. Vega F. E. Davis A. Borgemeister C. Chabi-Olaye A. (2011). Some like it hot: the influence and implications of climate change on coffee berry borer (Hypothenemus hampei) and coffee production in East Africa. PloS One 6, e24528. doi: 10.1371/journal.pone.0024528 21935419 PMC3173381

[B28] Jasso-Arreola Y. Ibarra J. A. Rosas-Cárdenas F. F. Estrada-De Los Santos P. (2025). Beneficial effects of ACC deaminase-producing rhizobacteria on the drought stress resistance of Coffea arabica L. Plants 14, 1084. doi: 10.3390/plants14071084 40219151 PMC11991408

[B29] Jiménez-Salgado T. Fuentes-Ramírez L. E. Tapia-Hernández A. Mascarúa-Esparza M. A. Martínez-Romero E. Caballero-Mellado J. (1997). Coffea arabica L. a new host plant for Acetobacter diazotrophicus, and isolation of other nitrogen-fixing acetobacteria. Appl. Environ. Microbiol. 63, 3676–3683. doi: 10.1128/AEM.63.9.3676-3683.1997 9293018 PMC168673

[B30] Jirakkakul J. Khoiri A. N. Duangfoo T. Dulsawat S. Sutheeworapong S. Petsong K. . (2023). Insights into the genome of Methylobacterium sp. NMS14P, a novel bacterium for growth promotion of maize, chili, and sugarcane. PloS One 18, e0281505. doi: 10.1371/journal.pone.0281505 36749783 PMC9904496

[B31] Johnson M. A. Ruiz-Diaz C. P. Manoukis N. C. Verle Rodrigues J. C. (2020). Coffee berry borer (Hypothenemus hampei), a global pest of coffee: perspectives from historical and recent invasions, and future priorities. Insects 11, 882. doi: 10.1111/jen.12804 33322763 PMC7763606

[B32] Johnston-Monje D. Gutiérrez J. P. Lopez-Lavalle L. A. B. (2021). Seed-transmitted bacteria and fungi dominate juvenile plant microbiomes. Front. Microbiol. 12, 737616. doi: 10.3389/fmicb.2021.737616 34745040 PMC8569520

[B33] Koutouleas A. Collinge D. B. Boa E. (2024). The coffee leaf rust pandemic: An ever-present danger to coffee production. Plant Pathol. 73, 522–534. doi: 10.1111/ppa.13846 40046247

[B34] Kunwar V. S. Chimouriya S. Lamichhane J. Gauchan D. P. (2018). Isolation and characterization of phosphate-solubilizing bacteria from rhizosphere of coffee plant and evaluating their effects on growth and development of coffee seedlings. Biotechnol. Indian J. 14, 1–10.

[B35] Kutos S. Bennett R. E. Santos D. Delgadillo E. B. Muletz-Wolz C. R. (2025). Soil and cherry bacterial communities predict flavor on coffee farms. Sci. Rep. 15, 19387. doi: 10.1038/s41598-025-03665-6 40461526 PMC12134262

[B36] Li L. Karunarathna S. C. Hyde K. D. Suwannarach N. Elgorban A. M. Stephenson S. L. . (2022). Endophytic fungi associated with coffee leaves in China exhibited *in vitro* antagonism against fungal and bacterial pathogens. J. Fungi 8, 698. doi: 10.3390/jof8070698 35887454 PMC9317674

[B37] Li S. J. Lei Y. X. Sun M. G. Liu H. F. Wang X. M. (2023). Research progress in the diversity of endophytic bacteria in seeds and their interaction with plants. Bio/Technol. Bull. 39, 166–175. doi: 10.13560/j.cnki.biotech.bull.1985.2022-0846

[B38] Lopez M. R. Cruz A. B. Lopez A. T. Bolaños T. A. (2025). Rhizospheric and endophytic plant growth-promoting bacteria associated with Coffea arabica L. and Coffea canephora Pierre ex Froehner: a review of their agronomic potential. Microorganisms 13, 2567. doi: 10.3390/microorganisms13112567 41304252 PMC12654060

[B39] Luiz B. C. Sugiyama L. S. Brill E. Keith L. M. (2024). Survey of potential fungal antagonists of coffee leaf rust (Hemileia vastatrix) on Coffea arabica in Hawai‘i, USA. Braz. J. Microbiol. 55, 2839–2844. doi: 10.1007/s42770-024-01304-2 38743246 PMC11405743

[B40] Mahatmanto T. Sunarharum W. B. Putri F. A. Susanto C. A. Davian A. O. Murdiyatmo U. (2023). The microbiology of Arabica and Robusta coffee cherries: a comparative study of indigenous bacteria with presumptive impact on coffee quality. FEMS Microbiol. Lett. 370, fnad024. doi: 10.1093/femsle/fnad024 37015877

[B41] Martinez S. J. Batista N. N. Bressani A. P. P. Dias D. R. Schwan R. F. (2022). Molecular, chemical, and sensory attributes fingerprinting of self-induced anaerobic fermented coffees from different altitudes and processing methods. Foods 11, 3945. doi: 10.3390/foods11243945 36553686 PMC9777685

[B42] Martins J. L. A. Franzin M. L. Ferreira D. D. S. Magina L. C. R. Martins E. F. Mendonça L. V. P. . (2024). Metarhizium-inoculated coffee seeds promote plant growth and biocontrol of coffee leaf miner. Microorganisms 12, 1845. doi: 10.3390/microorganisms12091845 39338519 PMC11433645

[B43] Mekete T. Hallmann J. Kiewnick S. Sikora R. (2009). Endophytic bacteria from Ethiopian coffee plants and their potential to antagonise Meloidogyne incognita. Nematology 11, 117–127. doi: 10.1163/156854108x398462

[B44] Mulaw T. B. Druzhinina I. S. Kubicek C. P. Atanasova L. (2013). Novel endophytic Trichoderma spp. isolated from healthy Coffea arabica roots are capable of controlling coffee tracheomycosis. Diversity 5, 750–766. doi: 10.3390/d5040750 30654563

[B45] Muleta D. Assefa F. Börjesson E. Granhall U. (2013). Phosphate-solubilising rhizobacteria associated with Coffea arabica L. in natural coffee forests of southwestern Ethiopia. J. Saudi Soc Agric. Sci. 12, 73–84. doi: 10.1016/j.jssas.2012.07.002 38826717

[B46] Numponsak T. Kumla J. Suwannarach N. Matsui K. Lumyong S. (2018). Biosynthetic pathway and optimal conditions for the production of indole-3-acetic acid by an endophytic fungus, Colletotrichum fructicola CMU-A109. PloS One 13, e0205070. doi: 10.1371/journal.pone.0205070 30335811 PMC6193638

[B47] Obieze C. C. Tapaça I. P. E. Graça I. De Melo Pereira G. V. Partelli F. L. Ramalho J. C. . (2025). Ecological function and diversity of the endosphere microbiome in leaves and fruits of Coffea arabica L. across elevation and shade gradients. Environ. DNA 7, e70119. doi: 10.1002/edn3.70119 41531421

[B48] Oliveira M. N. V. Santos T. M. A. Vale H. M. M. Delvaux J. C. Cordero A. P. Ferreira A. B. . (2013). Endophytic microbial diversity in coffee cherries of Coffea arabica from southeastern Brazil. Can. J. Microbiol. 59, 221–230. doi: 10.1201/9781315152769-19 23586745

[B49] Oliveira R. J. V. Souza R. G. Lima T. E. F. Cavalcanti M. A. Q. (2014). Endophytic fungal diversity in coffee leaves (Coffea arabica) cultivated using organic and conventional crop management systems. Mycosphere 5, 523–530. doi: 10.5943/mycosphere/5/4/4

[B51] Pereira T. S. Batista N. N. Pimenta L. P. S. Martinez S. J. Ribeiro L. S. Naves J. A. O. . (2022). Self-induced anaerobiosis coffee fermentation: impact on microbial communities, chemical composition and sensory quality of coffee. Food Microbiol. 103, 103962. doi: 10.1016/j.fm.2021.103962 35082079

[B50] Pereira C. M. Bautz K. R. Rodríguez M. D. C. Tobar L. M. S. Ndacnou M. K. Bekele K. B. . (2024). Cordyceps cateniannulata: an endophyte of coffee, a parasite of coffee leaf rust and a pathogen of coffee pests. Fungal Biol. 128, 1917–1932. doi: 10.1016/j.funbio.2024.05.004 39059847

[B52] Peterson S. W. Vega F. E. Posada F. Nagai C. (2005). Penicillium coffeae, a new endophytic species isolated from a coffee plant and its phylogenetic relationship to P. fellutanum, P. thiersii and P. brocae based on parsimony analysis of multilocus DNA sequences. Mycologia 97, 659–666. doi: 10.3852/mycologia.97.3.659 16392254

[B53] Pineda A. Dicke M. Pieterse C. M. J. Pozo M. J. (2013). Beneficial microbes in a changing environment: are they always helping plants to deal with insects? Funct. Ecol. 27, 574–586. doi: 10.1111/1365-2435.12050 40046247

[B54] Posada F. Aime M. C. Peterson S. W. Rehner S. A. Vega F. E. (2007). Inoculation of coffee plants with the fungal entomopathogen Beauveria bassiana (Ascomycota: Hypocreales). Mycol. Res. 111, 748–757. doi: 10.1016/j.mycres.2007.03.006 17604149

[B55] Pothakos V. De Vuyst L. Zhang S. J. De Bruyn F. Verce M. Torres J. . (2020). Temporal shotgun metagenomics of an Ecuadorian coffee fermentation process highlights the predominance of lactic acid bacteria. Curr. Res. Biotechnol. 2, 1–15. doi: 10.1016/j.crbiot.2020.02.001 38826717

[B56] Pratiwi E. R. Ardyati T. Suharjono S. (2020). Plant growth-promoting endophytic bacteria of Coffea canephora and Coffea arabica L. in UB Forest. J. Exp. Life. Sci. 10, 119–126. doi: 10.21776/ub.jels.2020.010.02.07

[B57] Ran X. L. Wei X. Y. Ren E. F. Qin J. F. Rasheed U. Chen G. L. (2025). Application of microbial fermentation in caffeine degradation and flavor modulation of coffee beans. Foods 14, 2606. doi: 10.3390/foods14152606 40807542 PMC12346622

[B58] Rosenblueth M. Martínez-Romero E. (2006). Bacterial endophytes and their interactions with hosts. Mol. Plant-Microbe Interact. 19, 827–837. doi: 10.1094/mpmi-19-0827 16903349

[B59] Saikai K. K. Oduori C. Situma E. Njoroge S. Murunde R. Kimenju J. W. . (2023). Biocontrol-based strategies for improving soil health and managing plant-parasitic nematodes in coffee production. Front. Plant Sci. 14, 1196171. doi: 10.3389/fpls.2023.1196171 37409284 PMC10319050

[B60] Sakiyama C. C. H. Paula E. M. Pereira P. C. Borges A. C. Silva D. O. (2001). Characterization of pectin lyase produced by an endophytic strain isolated from coffee cherries. Lett. Appl. Microbiol. 33, 117–121. doi: 10.1046/j.1472-765x.2001.00961.x 11472518

[B61] Santoyo G. Moreno-Hagelsieb G. Orozco-Mosqueda M. C. Glick B. R. (2016). Plant growth-promoting bacterial endophytes. Microbiol. Res. 183, 92–99. doi: 10.1016/j.micres.2015.11.008 26805622

[B62] Saucedo-García A. Anaya A. L. Espinosa-García F. J. González M. C. (2014). Diversity and communities of foliar endophytic fungi from different agroecosystems of Coffea arabica L. in two regions of Veracruz, Mexico. PloS One 9, e98454. doi: 10.1371/journal.pone.0098454 24887512 PMC4041768

[B63] Shen X. J. Wang Q. Wang H. S. Fang G. Q. Li Y. Zhang J. L. . (2024). Microbial characteristics and functions in coffee fermentation: a review. Fermentation 11, 5. doi: 10.3390/fermentation11010005 30654563

[B64] Shiomi H. F. Silva H. S. A. De Melo I. S. Nunes F. V. Bettiol W. (2006). Bioprospecting endophytic bacteria for biological control of coffee leaf rust. Sci. Agric. 63, 32–39. doi: 10.1590/s0103-90162006000100006 41099703

[B65] Silva H. S. A. Tozzi J. P. L. Terrasan C. R. F. Bettiol W. (2012). Endophytic microorganisms from coffee tissues as plant growth promoters and biocontrol agents of coffee leaf rust. Biol. Control 63, 62–67. doi: 10.1016/j.biocontrol.2012.06.005 38826717

[B66] Talhinhas P. Batista D. Diniz I. Vieira A. Silva D. N. Loureiro A. . (2017). The coffee leaf rust pathogen Hemileia vastatrix: one and a half centuries around the tropics. Mol. Plant Pathol. 18, 1039–1051. doi: 10.1111/mpp.12512 27885775 PMC6638270

[B67] Tenea G. N. Cifuentes V. Reyes P. Cevallos-Vallejos M. (2025). Unveiling the microbial signatures of Arabica coffee cherries: insights into ripeness-specific diversity, functional traits, and implications for quality and safety. Foods 14, 614. doi: 10.3390/foods14040614 40002058 PMC11854473

[B68] Teshome B. Wassie M. Abatneh E. (2017). Isolation, screening and biochemical characterization of phosphate-solubilizing rhizobacteria associated with Coffea arabica L. J. Fertil. Pestic. 8, 188. doi: 10.4172/2471-2728.1000188

[B69] Todhanakasem T. Ngo V. T. Pornpukdeewattana S. Charoenrat T. Young B. M. Wattanachaisaereekul S. (2024). The relationship between microbial communities in coffee fermentation and aroma with metabolite attributes of finished products. Foods 13, 2332. doi: 10.3390/foods13152332 39123524 PMC11312110

[B70] Trivedi P. Leach J. E. Tringe S. G. Sa T. Singh B. K. (2020). Plant–microbiome interactions: from community assembly to plant health. Nat. Rev. Microbiol. 18, 607–621. doi: 10.1038/s41579-020-0412-1 32788714

[B71] Truyens S. Weyens N. Cuypers A. Vangronsveld J. (2015). Bacterial seed endophytes: genera, vertical transmission and interaction with plants. Environ. Microbiol. Rep. 7, 40–50. doi: 10.1111/1758-2229.12181 40046247

[B72] Vaughan M. J. Mitchell T. McSpadden Gardener B. B. (2015). What’s inside that seed we brew? A new approach to mining the coffee microbiome. Appl. Environ. Microbiol. 81, 6518–6527. doi: 10.1128/aem.01933-15 26162877 PMC4561686

[B73] Vega F. E. Pava-Ripoll M. Posada F. Buyer J. S. (2005). Endophytic bacteria in Coffea arabica L. J. Basic Microbiol. 45, 371–380. doi: 10.1002/jobm.200410551 16187260

[B74] Vega F. E. Posada F. Aime M. C. Peterson S. W. Rehner S. A. (2008). Fungal endophytes in green coffee seeds. Mycosystema 27, 75–84.

[B75] Vega F. E. Simpkins A. Aime M. C. Posada F. Peterson S. W. Rehner S. A. . (2010). Fungal endophyte diversity in coffee plants from Colombia, Hawai‘i, Mexico and Puerto Rico. Fungal Ecol. 3, 122–138. doi: 10.4324/9781003249986-15

[B76] Wang B. T. Shi X. D. Shi M. N. Qi M. J. Zhang Z. W. Yang N. . (2024). Phyllospheric microorganisms and bean characteristics influence quality of ten genotypes of Coffea arabica. J. Soil Sci. Plant Nutr. 24, 3341–3354. doi: 10.1007/s42729-024-01757-2 30311153

[B77] Worku M. (2023). Production, productivity, quality and chemical composition of Ethiopian coffee. Cogent Food Agric. 9, 2196868. doi: 10.1080/23311932.2023.2196868 37339054

[B78] Wraight S. P. Howes R. L. Castrillo L. A. Griggs M. H. Wraight S. G. Carruthers R. I. . (2022). Laboratory studies assessing the microbial biocontrol potential of diverse strains of Beauveria bassiana isolated from coffee berry borer, with emphasis on strains from Hawai‘i Island and comparisons to commercial strain GHA. J. Invertebr. Pathol. 194, 107819. doi: 10.1016/j.jip.2022.107819 35987389

[B79] Wu Y. H. Zhao X. Wang Z. Q. Li X. J. Zhang X. S. Xie C. . (2025). The role of coffee microbiomes in pathogen resistance across varieties and ecological niches. Microorganisms 13, 1909. doi: 10.3390/microorganisms13081909 40871413 PMC12388573

[B80] Wulansari N. K. Prihatiningsih N. Utami D. R. Wiyantono W. Riyanto A. A. (2023). Isolation and identification of antagonistic fungi on coffee leaf rust in the Dieng highlands of Banjarnegara, Indonesia. Egypt. J. Biol. Pest Control 33, 72. doi: 10.1186/s41938-023-00718-8 38164791

[B81] Xiong C. Zhu Y. G. Wang J. T. Singh B. Han L. L. Shen J. P. . (2021). Host selection shapes crop microbiome assembly and network complexity. New Phytol. 229, 1091–1104. doi: 10.1111/nph.16890 32852792

[B82] Zhang C. L. Li X. J. Xie C. Chen Z. H. Lu Y. F. Wang B. T. . (2023). Effects of different slope aspects on Coffea arabica L. rhizospheric soil physical and chemical properties and microbial communities. J. South. Agric. 54, 3527–3537. doi: 10.22541/au.176055696.64184328/v1 42337277

[B83] Zhang J. Y. Zhao L. F. Liu M. J. (2025). Progress in research concerning the diversity, function, and application of plant endophytes. Acta Microbiol. Sin. 65, 1446–1468. doi: 10.13343/j.cnki.wsxb.20250012

[B84] Zhao S. G. Zhang A. Dong Y. P. Sun Y. Su L. Q. Lin X. J. . (2024). Intercropping with Areca catechu modulates rhizosphere microbial community structure and function to promote coffee plant development. Sci. Sin. Vitae 54, 1974–1987. doi: 10.1360/SSV-2023-0210

