# Supplementary material for: Coffee endophytes: diversity, ecological functions, and application prospects in sustainable production
Source: Front Plant Sci. 2026 Jul 7;17:1884416. doi: 10.3389/fpls.2026.1884416 (PMC13385118; doi:10.3389/fpls.2026.1884416)
Supplement: Supplementary file 1 [file Table1.docx]

Supplementary Material

**Supplementary Table 1** Representative coffee-associated microorganisms classified by tissue niche, ecological context, and evidence category

| **Microbial group/taxon** | **Tissue or niche** | **Coffee species/region** | **Method** | **Evidence category** | **Main inference** | **Limitation** | **Reference** |
| --- | --- | --- | --- | --- | --- | --- | --- |
| Bacterial endophytes including *Pseudomonas*, *Enterobacter*, *Acetobacter*, *Burkholderia*, *Bacillus*, and related taxa | Internal tissues, including stems, leaves, roots, and seeds | *Coffea arabica*/ not specified | Culture-dependent isolation from surface-sterilized tissues | Confirmed coffee endophyte | Coffee tissues harbor diverse culturable bacterial endophytes, including taxa with possible growth-promoting relevance | Mainly diversity/resource discovery; limited plant-level functional validation | Vega et al., 2005 |
| Nitrogen-fixing *Acetobacter* groups and other diazotrophic bacteria | Coffee tissues and rhizosphere | *C. arabica*/ Mexico | Isolation and nitrogen-fixation-related characterization | Coffee confirmed endophyte; coffee-associated rhizosphere isolate | Coffee plants can harbor diazotrophic bacteria in internal and root-associated niches | Quantitative contribution to coffee nitrogen nutrition remains unclear | Jiménez-Salgado et al., 1997 |
| Endophytic bacteria; selected isolates included TG4-Ia, TF2-IIc, TF9-Ia, TG11-IIa, and TF7-IIa | Leaves and branches/ internal tissues | *Coffea arabica and C. robusta* / São Paulo, Brazil | Endophyte isolation and rust biocontrol assays | Confirmed coffee endophyte | Some coffee endophytic bacteria reduced *Hemileia vastatrix* urediniospore germination and/or rust development | Controlled-condition evidence, field validation, persistence, mode of action, and biosafety need further study | Shiomi et al., 2006 |
| Endophytic bacteria and fungi; selected bacterial strains included 85G, 109G, 3F, 14F, 115G, 119G, and 137G | Leaves, branches, and roots/internal tissues | *Coffea arabica* and *C. robusta* / São Paulo, Brazil | Endophyte isolation and growth/rust assays | Confirmed coffee endophyte | Some bacterial endophytes promoted coffee seedling growth or reduced *Hemileia vastatrix* severity; fungal isolates were not effective | Controlled-condition evidence; no field validation; growth promotion and rust suppression were not linked in the same strains; biosafety requires further evaluation | Silva et al., 2012 |
| Root endophytic bacteria, including *Bacillus cereus* isolates SS.E2 and SW.E9 | Root endosphere | *C. canephora* and *C. arabica*/ Indonesia | Culture-dependent isolation and IAA assay | Confirmed coffee endophyte | Root endophytes may contribute to auxin-mediated growth regulation | Biosafety evaluation is required for *B. cereus*-related isolates | Pratiwi et al., 2020 |
| Endophytic bacterial isolates with phosphate solubilization, indole compounds, siderophores, HCN, and enzyme activities | Roots and seeds | Coffee plants/ Vietnam | Culture-dependent isolation and functional screening | Confirmed coffee endophyte | Coffee roots and seeds contain multifunctional bacterial endophytes with growth-promotion and antagonistic traits | Mostly in vitro screening; plant-level validation remains limited | Duong et al., 2021 |
| Phosphate-solubilizing bacteria; selected isolates included PSB 1, PSB 10, PSB 11, and PSB 20 | Rhizosphere of coffee seedlings | *Coffea arabica* / Khawa, Kavre, Nepal | Rhizosphere isolation, PSB screening, and seedling bioassays | Coffee-associated rhizosphere isolate | Coffee rhizosphere PSB improved seed germination and seedling growth; PSB 20 showed strong growth-promoting potential | Not confirmed as endophytes; isolates were not molecularly identified; greenhouse/controlled evidence only; field validation is needed | Kunwar et al., 2018 |
| Phosphate-solubilizing rhizobacteria, mainly *Pseudomonas*, *Burkholderia*, *Bacillus*, *Erwinia*, and related genera | Rhizosphere | *Coffea arabica* / Bonga and Yayu natural coffee forests, southwestern Ethiopia | Rhizobacterial screening and P-solubilization assays | Coffee-associated rhizosphere isolate | Coffee rhizosphere bacteria showed strong phosphate-solubilizing potential, mainly linked with medium acidification and organic acid production | Not confirmed as endophytes; in vitro nutrient-mobilization evidence only; plant-growth and field validation are needed | Muleta et al., 2013 |
| Phosphate-solubilizing bacteria, mainly *Pseudomonas*, *Bacillus*, *Enterobacter*, *Stenotrophomonas*, *Citrobacter*, and related genera | Rhizosphere soil, rhizoplane/root washing solution, and surface-sterilized roots | *Coffea arabica* / Kaffa and Jimma zones, southwestern Ethiopia | Isolation, biological identification, and P-solubilization assay | Coffee-associated rhizosphere isolate; confirmed root endophyte fraction | Coffee root-associated bacteria showed phosphate-solubilizing potential, with *Pseudomonas* as the dominant genus | Mixed niche evidence; mainly in vitro P-solubilization; no coffee plant-growth, colonization, or field validation | Teshome et al., 2017 |
| Root fungal endophytes, including diverse Ascomycota | Root endosphere | *C. arabica*/ Coffee-growing regions across climatic gradients | Sequencing/ endophyte community profiling | Confirmed coffee endophyte | Root endophyte communities vary across climatic gradients and host functional traits | Functional roles require experimental validation | Fulthorpe et al., 2020 |
| Fungal endophytes, mainly *Colletotrichum*, *Fusarium*, *Penicillium*, Xylariaceae, and other Ascomycota/Basidiomycota | Leaves, roots, stems, berries, seeds, crowns, and peduncles/internal tissues | Mainly *C. arabica*/ Colombia, Hawai‘i, Mexico, and Puerto Rico; other *Coffea* spp. also sampled in Hawai‘i | Endophyte isolation and ITS sequencing | Confirmed coffee endophyte | Coffee plants harbor highly diverse fungal endophyte communities that vary by region and tissue type | Culture-dependent study; plant age and shade conditions were not standardized; mostly diversity evidence without functional validation | Vega et al., 2010 |
| Foliar fungal endophytes, mainly Colletotrichum gloeosporioides complex, Phyllosticta capitalensis, Cladosporium, Xylaria, and related taxa | Leaf endosphere | *Coffea arabica* / Garanhuns, Pernambuco, Brazil; organic and conventional systems | Leaf endophyte isolation and morphological identification | Confirmed coffee endophyte | Organic and conventional coffee leaves harbored partly distinct fungal endophyte communities, with seasonal effects on abundance and richness | Culture-dependent and morphology-based identification; limited sampling; no functional validation; indicator species need confirmation | Oliveira et al., 2014 |
| Foliar fungal endophytes, mainly *Colletotrichum*, *Xylaria*, *Glomerella*, *Guignardia*, *Diplodia*, *Coniosporium*, *Paecilomyces*, and related taxa | Leaf endosphere | *Coffea arabica*/ Huatusco and Coatepec, Veracruz, Mexico; rustic plantations and simple polycultures | Leaf endophyte isolation and morphological/community analysis | Confirmed coffee endophyte | Foliar endophyte richness, diversity, and community composition varied with coffee region and, to a lesser extent, agroforestry system; rustic plantations tended to share more morphospecies | Culture-dependent and morphology-based identification; diversity was not fully recovered; functional roles require molecular and experimental validation | Saucedo-García et al., 2014 |
| Endophytic fungi in green coffee seeds | Seed endosphere | *C. arabica*/ Multiple origins | Culture-dependent isolation from surface-sterilized seeds | Confirmed seed endophyte | Coffee seeds can harbor fungal endophytes and may act as microbial reservoirs | Transmission, persistence, and functional roles need further validation | Vega et al., 2008 |
| Endophytic microbial communities in coffee cherries | Cherry/ fruit tissues | *C. arabica*/ Brazil | Culture-dependent and/or molecular microbial analysis | Fruit/seed-associated microbiota | Coffee cherries contain internal microbial communities relevant to fruit ecology and possible fermentation sources | Direct links with flavor formation require controlled validation | Oliveira et al., 2013 |
| Indigenous cherry-associated bacteria, including lactic acid bacteria, acetic acid bacteria, Enterobacteria, *Leuconostoc*, *Acetobacter*, *Bacillus*, *Pseudomonas*, and related taxa | Fresh coffee cherries / whole cherry-associated microbiota | *Coffea arabica* cv. Singosari and *C. canephora* cv. Sumbermanjing / Malang, East Java, Indonesia | Full-length 16S rRNA metagenome sequencing | Fruit/seed-associated microbiota | Arabica and robusta cherries harbored distinct indigenous bacterial communities; arabica was dominated by *Leuconostoc pseudomesenteroides*, while robusta showed higher bacterial diversity | Not confirmed as endophytes; whole-cherry sampling may include surface, internal, and environment-derived bacteria; findings are correlative and require isolation, localization, and fermentation/quality validation | Mahatmanto et al., 2023 |
| Fermentation-associated bacteria, mainly lactic acid bacteria, Enterobacter, Pseudomonas, Acetobacter, and related taxa | Fermented coffee cherries / recovered microbial biomass | *Coffea arabica* varieties/groups Typica, Yellow Caturra, and Red Caturra / Intag Valley, Imbabura, Ecuador; green and ripe cherry stages | Shotgun metagenomic sequencing and functional annotation | Fermentation-associated microbiota | Coffee variety, ripeness stage, and fermentation context shaped microbial diversity and functional profiles, with implications for quality and safety | Not confirmed as endophytes; fermentation microbiota may include fruit-, surface-, and environment-derived microbes; findings are mainly metagenomic/correlative and need isolation, sensory, and controlled fermentation validation | Tenea et al., 2025 |
| Leaf- and fruit-endosphere bacteria and fungi | Leaves and fruits | *C. arabica*/ Mozambique | Endosphere microbiome sequencing | Confirmed coffee endophyte | Altitude and shade influence bacterial and fungal endosphere communities | Functional predictions need experimental confirmation | Obieze et al., 2025 |
| Fungi associated with rust lesions or diseased leaf microhabitats, including *Simplicillium*, *Cladosporium*, and *Trichoderma* | Rust lesions, diseased leaves, or phyllosphere-associated material | *C. arabica*/ Hawai‘i and Indonesia | Isolation and antagonism assays | Coffee-associated phyllosphere/lesion isolate | Lesion-associated fungi may provide local antagonists against *Hemileia vastatrix* | Should not be treated as confirmed endophytes unless internal colonization is demonstrated | Luiz et al., 2024; Wulansari et al., 2023 |
| Coffee-associated bacterial communities, including cherry- and soil-associated taxa such as Proteobacteria, Actinobacteriota, Acidobacteriota, Firmicutes, and related ASVs | Coffee cherries and farm soil | *Coffea arabica* / San Francisco, Cundinamarca, Colombia; shade and sun farms; different flavor categories and harvest periods | 16S rRNA amplicon sequencing, soil analysis, and flavor/management comparison | Fruit/seed-associated microbiota; coffee-associated soil microbiota | Cherry and soil bacterial communities varied with harvest period, shade management, and flavor category, suggesting that local farm microbiomes may contribute to coffee flavor differentiation | Not confirmed as endophytes; cherry samples may include surface, internal, and environment-derived bacteria; bacterial-only sequencing; correlative evidence without strain isolation or causal flavor validation | Kutos et al., 2025 |
| Fermentation-associated bacteria and yeasts, including *Lactiplantibacillus plantarum*, *Leuconostoc mesenteroides*, *Weissella cibaria*, *Pediococcus pentosaceus*, *Hanseniaspora uvarum*, *Saccharomyces cerevisiae*, and related taxa | Natural and pulped coffee fermentation mass / postharvest processing environment | *Coffea arabica* / Monte Carmelo, Três Pontas, Carmo de Minas, and Lajinha, Minas Gerais, Brazil | Culture-dependent isolation, MALDI-TOF/sequence identification, NGS, NMR, and sensory analysis | Fermentation-associated microbiota | Self-induced anaerobiosis altered microbial communities, chemical profiles, and sensory attributes, especially intensifying fruity notes in fermented coffee | Not confirmed as endophytes; microbiota may include fruit-surface, pulp/mucilage, processing-environment, and fermentation-derived microbes; causal roles require controlled inoculation and validation | Pereira et al., 2022 |
| Fermentation-associated bacteria, yeasts, and fungi, including *Leuconostoc, Lactococcus, Lactobacillus, Pseudomonas, Enterobacter, Weissella, Cladosporium, Pichia, Papiliotrema,* and *Rhodotorula* | Wet-fermented coffee cherries and fermentation slurry | *Coffea arabica* / Chiang Mai, Thailand | 16S/ITS amplicon sequencing with volatile and metabolite profiling | Fermentation-associated microbiota | Microbial succession during wet fermentation was associated with changes in aroma compounds and metabolites, suggesting possible roles in coffee flavor development | Not confirmed as endophytes; fermentation communities may include fruit-, surface-, water-, equipment-, and environment-derived microbes; correlations need controlled inoculation and sensory validation | Todhanakasem et al., 2024 |

**Note:** Evidence categories were used to distinguish confirmed coffee endophytes from coffee-associated rhizosphere isolates, phyllosphere/lesion isolates, fruit/seed-associated microbiota, and fermentation-associated microbiota.
